# Evaluation of indicators of acute emotional states in dogs

**DOI:** 10.1038/s41598-024-56859-9

**Published:** 2024-03-17

**Authors:** Hannah E. Flint, Jennifer E. Weller, Nia Parry-Howells, Zack W. Ellerby, Stephanie L. McKay, Tammie King

**Affiliations:** Waltham Petcare Science Institute, Waltham on the Wolds, LE14 4RT UK

**Keywords:** Animal behaviour, Animal physiology

## Abstract

A complete assessment of animal welfare requires not just an understanding of negative emotional states, such as fear and anxiety, but also of positive states, such as calmness and happiness. However, few studies have identified accurate and reliable indicators of positive emotional states in dogs. This study aimed to identify parameters that may serve as indicators of short-term emotional states in dogs. Using a cross-over design, 60 dogs living at a research facility were exposed to six different 10-min scenarios expected to elicit responses varying in emotional valence and arousal. A range of behavioural and physiological parameters were collected and their relationship to anticipated emotional valence and arousal was analysed using linear and logistic mixed models. Cortisol, adrenocorticotropic hormone, heart rate variability, panting, whining, and body shake all demonstrated significant differences based on arousal levels, but only within negative valence scenarios. Scores from a qualitative behavioural assessment (QBA) were associated with both emotional valence and arousal and were considered the best indicator of positive valence. Activity, ear temperature, and sitting were associated with positive high arousal, although this may have been influenced by differing levels of movement induced during these scenarios. Meanwhile, heart rate, secretory immunoglobulin A, standing and lying all showed similar changes associated with arousal for both positive and negative valence scenarios. This study provides a critical first step towards identifying evidence-based indicators of short-term emotional states in dogs, while highlighting considerations that should be made when employing these parameters, including the influence of coder bias, food provision, exercise, and external temperature. Overall, it is recommended future dog emotion and welfare research use a combination of parameters including indicators of both emotional valence and arousal.

## Introduction

In recent years, there has been a growing interest in the accurate assessment of dog welfare^1–6^. It is widely recognized in the field of animal welfare science that the absence of negative emotions is not enough to constitute good welfare^7–9^, however, most of the research conducted in this area has focused on assessing and minimizing negative emotional states, such as fear and anxiety, e.g.^10–16^. Little research has been conducted on the assessment or promotion of positive emotions, such as joy and happiness^3,7,17^. This is potentially due to the subtle expression of such emotional states compared to their negative counterparts. Subsequently, a consensus on how to assess the positive emotions experienced by animals has yet to be reached^7^ and no single indicator of positive emotion in dogs has been validated^3^. Despite this, there is universal agreement that accurate and reliable indicators of both positive and negative emotional states are required to accurately assess dog emotional wellbeing and the impact it has on welfare.

**Figure 1:**
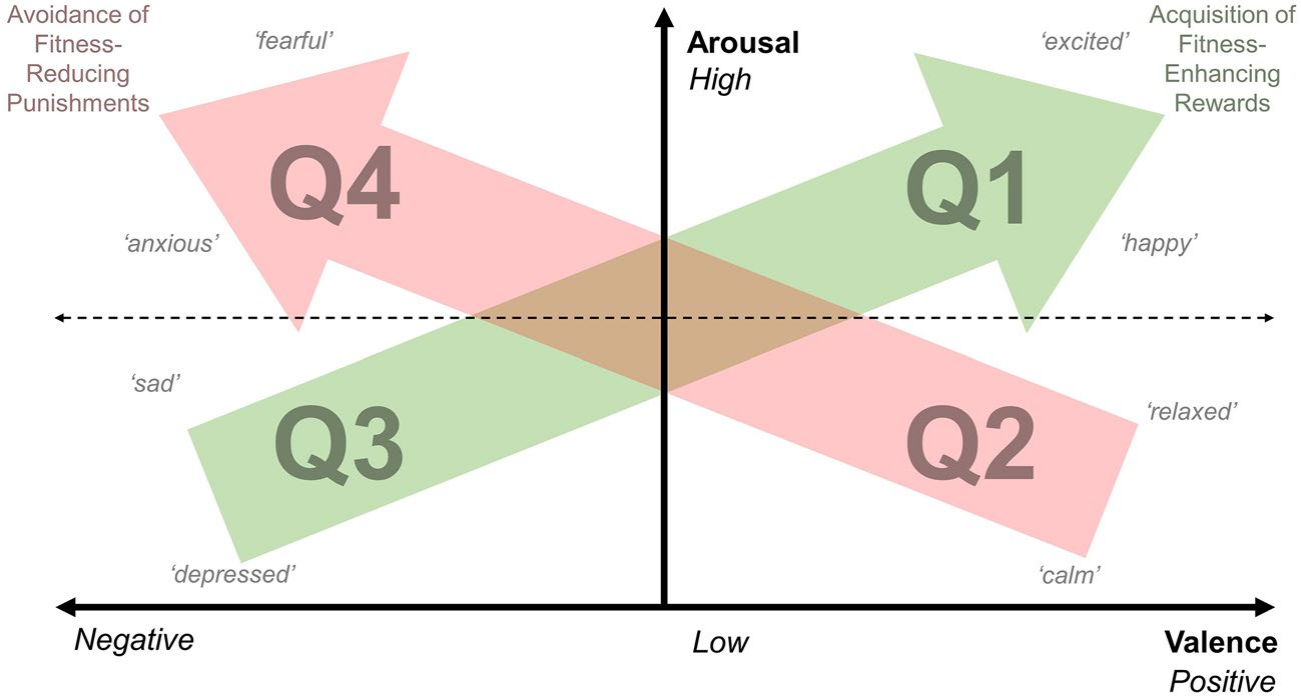
Core affect represented as a two-dimensional space along both a valence (x) and arousal (y) axis. Quadrants Q1 and Q2 represent positive affective states with high and low arousal respectively. Quadrants Q3 and Q4 represent negative affective states with low and high arousal respectively. Arrows indicate the adaptive emotional systems related to the acquisition of fitness-enhancing rewards (green) and the avoidance of fitness-reducing punishments (red). Words in grey italics indicate possible locations of specific discrete emotions within the core affect. Adapted from Mendl et al.^19^.

One common theory is that emotions exist across two dimensions, the first being a dimension of valence leading from positive to negative, and the other being a dimension of activation/arousal ranging from energy conserving to active^18–20^. Such an approach allows the visualization of discrete emotions within a two-dimensional space (Fig. 1), and results in the formation of four quadrants each representing a different aspect of core affect: (1) Positive/High arousal, (2) Positive/Low arousal, (3) Negative/Low arousal, (4) Negative/High arousal. The first and third quadrants contain positive/high arousal emotions, such as joy, and negative/low arousal emotions, such as sadness, suggesting that emotional states located along the Q3–Q1 axis are related to the acquisition of fitness-enhancing rewards. The second and fourth quadrants contain positive/low arousal emotions, such as calmness, and negative/high arousal emotions, such as fear, suggesting that emotional states located along the Q2–Q4 axis are related to the absence of fitness-reducing punishments. It therefore can be anticipated that by manipulating the presence or absence of different environmental stimuli (relating to reward or threat), emotions from the different quadrants can be successfully induced.

While it is not possible to directly assess the conscious experience of emotion, several measurable physiological and behavioural indicators of emotional states have been suggested^3,19^. However, their ability to indicate positive emotions has been widely untested. Physiological indicators of emotion are primarily related to the relative activation of different neuroendocrine systems, such as the autonomic nervous system (ANS) and the hypothalamic–pituitary–adrenal axis (HPA)^3,7^. On the other hand, behavioural metrics can focus on absolute quantitative parameters, such as the duration of time spent performing a specified behaviour, or qualitative parameters, which utilize a ‘whole animal’ perspective to determine not what behaviours an individual is performing, but the demeanor with which they are performing them^21–23^. The general way in which an individual expresses a series of behaviours can be captured using Qualitative Behavioural Assessment (QBA), which typically requires a rater to observe an individual animal over a predetermined period of time. Raters are then asked to score the emotional expressivity of the subject’s behaviour on either a set of predefined or free-choice terms, such as ‘Relaxed’, ‘Nervous’, or ‘Curious’^22,24^. These scores are then typically refined into multiple component scores, representing groups of related terms, through means of Principle Component Analysis (PCA). QBA has been utilized in the welfare assessment of wide variety of species (e.g., cows^25^, pigs^26^, sheep^27^, horses^28^, donkeys^29^, goats^30^, chickens^24^, dogs^22^, polar bears^31^, elephants^32^, dolphins^33^, salmon^34^), as it is often suggested that included terms (such as ‘Playful’ or ‘Relaxed’) can be representative of an animal’s emotional state^7,26^. However, like all measurement tools, the process of continued validation is critical^26^ and research exploring the validity of QBA as a reliable indicator of valence and arousal appears to have been limited. For example, a recent study by Skovlund et al*.*^31^ found that constructs of valence and arousal obtained from QBA were associated with additional animal-based welfare indicators in captive polar bears, although these indicators have not themselves been fully validated.

Subsequently, the primary aim of this study was to identify physiological and behavioural parameters (or a combination of parameters) that may serve as reliable indicators of short-term emotional valence and/or arousal in dogs. Based on previous literature, a total of five potential parameters were initially selected for investigation; heart rate (HR), heart rate variability (HRV) measured as the root mean square of successive differences in the RR interval (RMSSD), two putative QBA component scores expected to represent valence and arousal^24,31,35^, and blood serum cortisol. The secondary aim of this study was to explore a further 17 additional parameters; blood serum serotonin, blood plasma adrenocorticotropic hormone (ACTH), salivary secretory immunoglobulin A (sIgA), HRV measured using the standard deviation of the RR interval (SDRR), activity score, multiple eye, ear, and nose temperatures, body position, panting, body shake, and whining, which were also predicted to be impacted by an individual’s emotional state.

Lastly, this study aimed to explore the effect of providing food on these parameters, as previous studies have indicated that the relationship between some of these parameters and dogs’ emotional states will be influenced by provision of food. For example, Kostarczyk and Fonberg^36^ observed that the process of eating can induce periods of cardiac accelerations and deceleration, with the reinforcing value of the consumed food impacting the pattern of HR. Furthermore, the provision of food has been suggested to impact both the feasibility and reliability of salivary sampling^37^. Given that food is often employed during the simulation of positive scenarios, it is fundamentally important to determine how this may impact indicators of emotional state outside of the context of increased valence.

## Methods

### Pilot study

To identify suitable physiological and behavioural parameters for the assessment of emotional states in dogs, an a priori approach was adopted^19^ to select scenarios that generated emotional states within each of the four core affect quadrants. A pilot study utilizing 20 adult dogs (8 males, 12 females; 5 Labrador Retrievers, 5 Beagles, 5 Norfolk Terriers, and 5 Petite Basset Griffon Vendéens) was conducted. Dogs were housed in pairs or groups of three within kennels located at the Waltham Petcare Science Institute (Leicestershire, UK), which allowed for free access to both an indoor and outdoor environment. Throughout the study, all dogs experienced comprehensive training and socialization programs as per the Institute’s standard animal care requirements. Additionally, dogs were habituated to all testing environments and associated equipment prior to testing.

**Figure 2:**
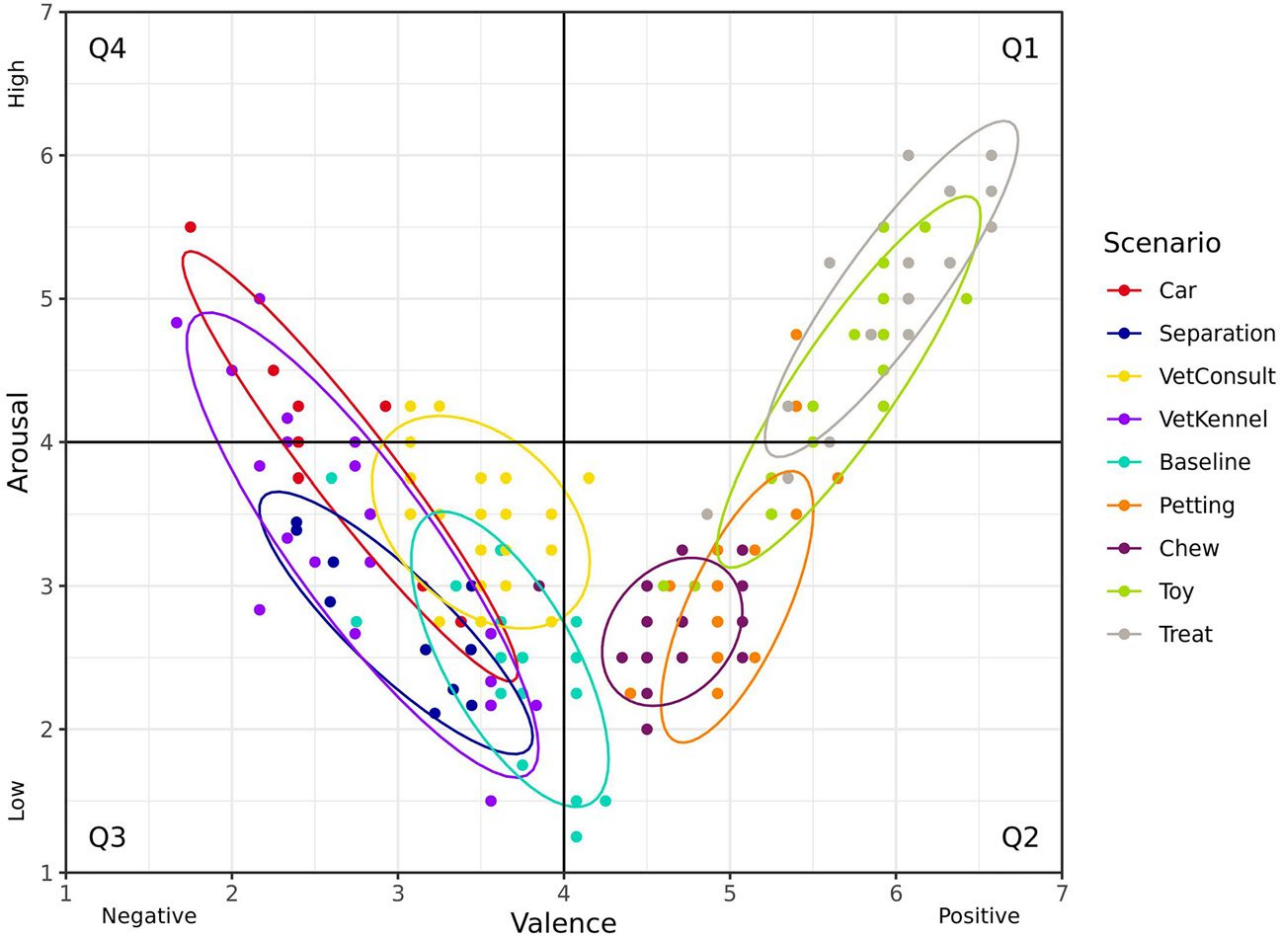
Dogs’ responses to different scenarios anticipated to elicit emotions varying from positive to negative valence and low to high arousal during the pilot study. Individual dog responses are indicated by mean valence and arousal ratings provided by two trained dog behaviour coders, with 80% confidence (data) ellipses for each scenario. Valence was scored on a scale from 1 (very negative) to 7 (very positive), and arousal scored from 1 (very low) to 7 (very high), with the midpoint (4) indicated.

The dogs were exposed to four scenarios anticipated to induce positive valence emotions: provision of a long-lasting chew (chew); calm petting by a familiar handler (petting); engaging in play with a toy (toy); engaging in a game throwing treats (treat)*.* Additionally, video footage from previous research exploring five different scenarios anticipated to induce negative valence emotions was reviewed: confinement to the inside portion of their home enclosure whilst isolated from conspecifics (baseline^38^); social isolation in a familiar room (separation^38^); housed in a kennel in a vet suite (kennel^39^); a veterinary examination (consult^39^); and car travel (car^38^). Video recordings of dogs experiencing these scenarios were scored by two trained dog behaviour coders on a scale of one to seven for valence (1—very negative, 7—very positive) and arousal (1—no arousal, 7—high arousal) in order to assess if the emotional state induced fell within the required quadrant for a majority of dogs tested. In instances where a scenario exceeded 10 min, only the first 10 min were scored. Four scenarios (two utilizing a food reward and two utilizing a social reward) resulting in emotional arousal and valence characteristic of Q1 and Q2 were selected. Additionally, two scenarios suitable for eliciting emotional responses consistent with Q3 and Q4 were also identified. These scenarios were selected based on the percentage of dogs that fell within the defined emotional quadrant. Further considerations resulted in selection of sessions that ensured the highest level of separation between the different quadrants (Fig. 2).

The chew and treat scenarios were selected to induce emotions in the presence of food within Q2 (positive valence/low arousal) and Q1 (positive valence/high arousal) respectively, while toy and petting scenarios were chosen to induce Q2 and Q1 emotions without the presence of food. Emotions in Q3 (negative valence/low arousal) and Q4 (negative valence/high arousal) were induced using the separation and car scenarios respectively.

### Statistical powering

The sample size for this study was determined through a priori power analysis by simulation, for each primary measure of interest (Cortisol, HR, HRV-RMSSD, both QBA component scores). Plausible effect sizes and within- and between-animal variance components were estimated and/or extrapolated from a subset of existing data (control diet, pre- and post- first exposure) collected in previous research measuring the same parameters in negative emotion settings only^38^. These values were used to simulate 1000 datasets in the proposed experimental design (2 × 2 crossover), for each primary measure and at each of a range of potential sample sizes. Each simulated dataset was analyzed according to the planned statistical approach and pairwise contrast design for the main study (see below), and the proportion of simulations in which induced pairwise effects of interest were detected was recorded for each measure as an empirical power estimate. Based on the results of these analyses a sample size of 60 dogs was chosen for the main study in order to achieve power exceeding 80% to detect a difference relating to valence and/or arousal for each of the five primary measures. This assumed that residual variability and effect sizes of interest would not be substantially greater or smaller in positive valence conditions, respectively—an assumption that was generally borne out in our subsequent data, entailing no major concerns around statistical power for this study.

### Animals and husbandry

Upon completion of the pilot study, 60 healthy, adult dogs consisting of 31 males (4 entire) and 29 females (7 entire) from three breeds (30 Labrador Retrievers, 12 Beagles, 18 Norfolk Terriers) participated in the main study. Dogs ranged in age from 0.9 to 6.1 years old (mean age = 3.2) at the start of the study. Dogs varied in their experience with the selected scenarios based on their training history and previous participation in research studies. Dogs for the main study were housed, managed, and habituated in the same manner as the pilot dogs. Additional training to facilitate sample collection (i.e., blood draws, mouth and ear handling, wearing multi-parameter harnesses) was also conducted prior to testing. All dogs visited the room used for test sessions a minimum of two times prior to the start of the study, with additional visits provided if the dog showed signs of negative emotional reactions (e.g., fear, anxiety) or high arousal positive emotional reactions (e.g., excitement, anticipation). Visits were combined with the dog’s usual daily exercise and included being let off-lead in the test room for a few minutes. Dogs were able to freely investigate the area, and the handlers were instructed not to encourage the dogs using food, toys, or play in order to minimize the dogs developing strong positive or negative associations with the space. Further, all dogs were taken to the test room for at least one recovery session (with additional sessions provided if negative emotional reactions observed) in between test sessions in order to minimize the impact of previous scenarios on the dogs’ responses to entering the room. All dogs were trained to walk up or onto a ramp or box (based on the dog’s individual preference) to enter the car and into a crate fixed inside the car. The number of training sessions provided to dogs was based on the emotional reaction and training progression of the individual dog. All dogs were required to be comfortable and willing to enter the crate in the car without strong positive or negative emotional reactions (e.g., extremely excited or nervous) prior to their car test session.

Dogs were excluded from the study if they failed to adequately habituate to these sample collections or test areas prior to the start of the study, and replacement dogs were selected. Additionally, for the purposes of dog and human safety, dogs were excluded based on previous observations of excessive destructive behaviour, or resource guarding, as well as any dietary restrictions that would not allow the consumption of the treats used during testing.

This study was approved by the Waltham Animal Welfare and Ethical Review Body (WAL 102424) and conducted under the authority of the UK Animals (Scientific Procedures) Act 1986. All methods were performed in accordance with relevant guidelines and regulations and are reported in accordance with ARRIVE guidelines.

During each test session dogs were closely monitored through means of live-feed CCTV cameras (Dahua 4K IR Turret Network Camera; Dahua Technology, Leeds, UK). Dogs were monitored for signs of distress and/or safety concerns based upon predefined end-point criteria. These included hyperventilation, extreme hypersalivation, excessive barking or whining, cowering, repeated performance of vigorous escape attempts, and behaviours that had the potential to result in self-harm and/or the ingestion of a foreign body. No dogs had to be removed from the study due to signs of distress, however, one dog’s car scenario was terminated early due to unrelated mechanical issues with the car. Additionally, one male Labrador was removed from the study (and therefore any subsequent analyses) after being diagnosed with atypical Addison’s disease.

### Study design

Each dog was exposed to each of the six selected scenarios over a period of 19 weeks using a cross-over study design with order randomized based on a balanced Latin square. Test sessions were scheduled 3 weeks apart with some exceptions due to scheduling conflicts (min. 12 days). In order to minimize the potential impact of routine vaccinations and/or certain medications on the immunological parameters collected, dogs skipped sessions within 4 weeks of the administration of these substances, resulting in up to 6 weeks between sessions. To maintain balanced ordering, missed sessions were rescheduled to the next time-slot and remaining sessions pushed backwards in turn, with the end of the study slightly delayed for these animals. Due to the nature of the test sessions used, the handlers and experimenters were not able to be blinded.

All scenarios lasted 10 min and, with the exception of car travel, occurred within a test room (5.23 m × 3.68 m) which the dogs had been previously habituated to. The test room contained multiple resting areas (two pieces of vet bed on the floor and a piece of vet bed on an elevated platform) and fresh water (water bowl that was emptied and re-filled at the beginning of each test session). To mask potentially inconsistent background noises that might distract dogs during the scenarios, a radio was played either directly outside the test room or through the car speakers, set to a consistent volume and radio station. To minimize the effect of external temperature all testing and sampling areas were maintained at 18 ± 2 °C. Throughout all testing and sampling procedures each dog was handled by an individual who regularly worked with and trained that dog. This resulted in different handlers being used for different dogs, as appropriate. The research team, including authors (S.L.M), oversaw the test sessions, but were not directly involved in handling the dogs.

The six scenarios utilized in the main study to elicit various emotional states in dogs are outlined in detail below:

#### Positive valence/high arousal/with food—treat throwing

The dog was taken into the test room by a familiar handler, the lead removed, and given 2 min to acclimate and explore. The handler then retrieved a container of pre-prepared treats (CRAVE™ Protein Chunks; Mars Petcare, Slough, UK) from a shelf and sat on a chair located in a corner of the room. The number of treats prepared for this scenario was determined based on the individual weight of the dog being tested, with dogs over 25 kg receiving 18 chunks, dogs between 10 and 25 kg receiving 13 chunks, and dogs under 10 kg receiving 8 chunks. These chunks were then cut into smaller pieces so that each dog had a total of 72 treat pieces available for throwing. The handler took single treat pieces from the container and threw them in random directions and distances, utilizing the entire test room. Treat pieces were thrown approximately once every 5–10 s. The handler could speak to the dog as required to engage them in the game. After 10-min the handler re-attached the lead and walked the dog to an adjacent room for post-test sampling.

#### Positive valence/high arousal/without food—toy play

The dog was taken into the test room by a familiar handler, the lead removed, and given 2 min to acclimate and explore. The handler then engaged the dog in play with a selection of toys for 10-min. All dogs had exposure to a range of toys prior to testing, and their top two preferred toys were used during testing. Handlers were instructed to engage the dogs in their preferred style that maximised engagement and excitement. This could include fetch, tug or chase style games. After 10-min the handler retrieved the toys, re-attached the lead, and led the dog to an adjacent room for post-test sampling.

#### Positive valence/low arousal/with food—long lasting chew

The dog was taken into the test room by a familiar handler, the lead removed, and given 2 min to acclimate and explore. The handler then retrieved a long-lasting chew (PEDIGREE© GOOD CHEW™ Treat, Mars Petcare UK, Slough, UK) from a shelf and placed it on the floor in the middle of the room. Dogs were provided with an appropriately sized chew based on the dog’s weight, with dogs over 25 kg receiving a large chew, dogs between 10 and 25 kg receiving a medium chew, and dogs under 10 kg receiving a medium chew cut in half lengthwise. To minimize the handler inadvertently distracting the dog during this scenario, the handler remained within the test room, sat on a chair in a corner of the room, and occupied themselves with a digital tablet kept on silent. However, if the dog solicited attention during the test session the handler was permitted to calmly acknowledge the dog and direct the dog’s attention back to the chew if not yet consumed. After 10-min the handler re-attached the lead and led the dog to an adjacent room for post-test sampling.

#### Positive valence/low arousal/without food—petting

The dog was taken into the test room by a familiar handler, the lead removed, and given 2 min to acclimate and explore. The handler then sat on vet bedding, which was placed on the floor, and gently encouraged the dog to come close. The handler then stroked or scratched the dog in a calming or soothing manner, based on the dog’s individual preferences. Handlers were instructed to halt and/or alter their approach if the dog showed signs of excessive excitement or discomfort (e.g., yawning, panting, moving away). If dogs became disengaged from the handler and moved out of reach, the handler periodically encouraged them to return, but the dogs were otherwise allowed free choice whether to continue the interaction. After 10-min the handler re-attached the lead and led the dog to an adjacent room for post-test sampling.

#### Negative valence/high arousal—car travel

Dogs were walked on lead by their handlers, to a minivan vehicle (Ford S-MAX; Ford Motor Company Ltd., Essex, UK) parked outside the post-test sampling room. Dogs entered the rear of the car via a ramp or platform (depending on the dogs predetermined preference) and were closed within a crate secured within the car boot. The size of the crate used was dependent on the size of the dog (small crate: 76 × 48 × 54 cm, medium crate: 78 × 54 × 62 cm, large crate: 90 × 58 × 66 cm, XL crate: 106 × 71 × 70 cm), and each crate contained a piece of non-slip vet bedding. The car then underwent a standardized 10-min car journey consisting of a range of maneuvers including a sharp U-turn and a three-point-turn. The speed of the car never exceeded 10 mph due to being in a private enclosed car park area. Upon completion of the route, the handler opened the car boot and crate, re-attached the lead, and led the dog out of the car via the ramp or platform and into the building for post-test sampling.

#### Negative valence/low arousal—separation

The dog was taken into the test room by a familiar handler, the lead removed, and given 2 min to acclimate and explore. The handler then left the room, and the dog was left alone for a period of 10-min while being monitored by a researcher in an adjacent room via a CCTV system. After 10-min the handler returned, re-attached the lead, and led the dog to an adjacent room for post-test sampling.

### Data collection and processing

A range of behavioural and physiological parameters were captured during and after testing to determine which parameters, or combination of parameters, could be successfully utilized to differentiate between different emotional states. These parameters included data generated during the test sessions from wearable devices worn by the dog, and behavioural data coded from video footage. After test sessions, dogs were taken from the testing area to a room for post-test sampling. Prior to entry to the sampling room, infra-red videos were collected for measurement of surface body temperature of key areas of the dog. Upon entry to the sampling room, tympanic temperatures were collected, followed by blood samples for measurement of cortisol, serotonin and ACTH, and saliva samples for measurement of sIgA. Further details related to the collection and processing of these parameters are outlined below.

#### Wearable technology parameters

Two different wearable technologies were used to measure a range of parameters during test sessions. These included activity monitors (Whistle™ FIT accelerometer; Mars Petcare, McLean, VA, USA) which have been previously validated for collection of activity data^40^, and multi-parameter harnesses (Dinbeat UNO; Dindog Tech, S.L., Barcelona, Spain) which have been previously validated for collection of HR and HRV data^41^ and also provided readings for body position (unvalidated).

The activity monitors were attached to the dog’s collar and worn throughout testing. One minute Activity Points generated by the activity monitor indicative of duration and intensity of activity during that time period were matched to the test session times and summarized to determine mean Activity Points during the test session.

For the multi-parameter harnesses, on the day prior to testing, dogs had their fur clipped in three specific areas on the sides of their chest (one area on either side of their rib cage about an inch from their arm pit and one area on their right side towards the end of their rib cage) to allow for the application of electrocardiogram (ECG) electrodes. On the day of testing, dogs were equipped with the multi-parameter harness which was worn throughout testing.

Following testing, data were downloaded from the devices, which consisted of HR (bpm) and categorical position readings (standing, sitting, lying sternal, lying left lateral, lying right lateral, supine, on two legs) provided 24 times per second. RR intervals (ms) based on continuous ECG data were also obtained. These data were matched to the test session times and summarized to determine mean HR and proportion of time spent in each position during the test session. Additionally, HRV was calculated as the root mean square of successive RR interval differences (RMSSD) as well as the standard deviation of the RR intervals (SDRR). A single HRV value was generated for both RMSSD and SDRR for each 10-min test session. As HR readings occasionally dropped out when ECG nodes moved, or the device lost connection, any sessions with more than 50% missing readings for HR or RR interval were excluded from analysis (n = 32). Furthermore, a total of five dogs did not wear the multi-parameter harness due to failure to successfully habituate to the device, as demonstrated by alterations to their normal behaviour. Also, 40 videos (11.4%) were randomly selected to be coded by a trained dog behaviour coder and used to assess agreement between the harness readings and manual coding (Table 1). The appropriate number of videos to assess reliability was determined from a review of literature on sample size requirements for reliability analyses based on assumed moderate to good agreement (ICC ~ 0.60)^42–44^. However, three videos could not be compared to corresponding Dinbeat harness readings due to data not being available from the harnesses for that session. For the purposes of comparison and analysis, lying sternal, left lateral, right lateral, and supine as measured by the multi-parameter harness were combined for a total proportion of time lying, sitting was used to determine proportion of time sitting, and standing and on two legs were combined for a total proportion of time standing. Meanwhile, the video coded behaviours of lateral lie down and sternal lie down were combined for a total proportion of time lying, sit was used to determine proportion of time sitting, and stand, walking, trotting and vigorous activity were combined for a total proportion of time standing.

**Table 1:** Behavioural terms and definitions used to assess dog position and activity during test sessions.

Term	Definition
Lateral Lie Down	Body in contact with ground not supported by legs, side of dog touching the ground/bedding fully^45^
Sternal Lie Down	Body in contact with ground not supported by legs, sternum touching ground/bedding and hind limbs on either side^45^
Sit	Front legs straight, rear end lowered and resting on hocks^45^
Stand	Hind two paws or all four paws on ground and legs upright and extended supporting body^46^
Walking	Four beat gait, three feet on the ground at any one time^45^
Trotting	Two beat gait, diagonally opposite legs move together^45^
Vigorous Activity	Rapid/energetic movement in any direction, including running, bounding, tugging, jumping, rolling around (defined for this study)

#### Video parameters

Video footage for coding of dog behaviour data were collected via four CCTV video cameras mounted in each corner of the room for scenarios conducted within the test room. During the car scenario, video footage was recorded using two Logitech 922 webcams (Logitech, Lausanne, Switzerland) which were mounted with a view of the front (on car center console) and rear (on rear car window) of the crate.

Videos from each 10-min test session were coded for a number of behaviours anticipated to vary based on emotional state using a detailed ethogram (Table 2). One trained dog behaviour coder scored all videos using *‘The Observer XT 15*’ (Noldus, Netherlands, Europe). Further, a random selection of 10 videos (2.9%) were re-coded by the same coder for a total of three repetitions, with repeats randomly distributed throughout the course of data collection, to assess intra-rater reliability. The appropriate number of videos to assess intra-rater reliability was determined from a review of literature on sample size requirements for reliability analyses based on assumed good to excellent agreement (ICC ~ 0.80)^42–44^. Video names were encoded so that the coder was blind to which videos were repetitions. To account for minor differences in video length, state behaviours were analyzed as a proportion of time spent performing the behaviour by dividing the duration of the behaviour by the total video length. Further, due to the mouth of the dog not always being visible from the available camera angles, the proportion of time spent panting was divided by the duration of the video where the mouth was visible. Videos (n = 18) where the mouth was not visible for more than 25% of the video duration were not included in the analysis of panting behaviour.

**Table 2:** Behavioural terms and definitions used to assess dog emotion during test sessions.

Term	Definition
Whining	Dog produces sounds such as whines, whimpers, yelps, etc. originating from the throat and mouth^38^
Not Whining	Sound production ceases^38^
Panting	Increased shallow respiration through an open mouth, may have tongue out^47^
Mouth not visible	Can not see mouth area. Panting/Not Panting cannot be determined (*defined for this study*)
Not Panting	Mouth is closed—normal breathing resumes^38^
Yawn	An involuntary intake of breath through a wide-open mouth^38^
Shake	Dog's whole body and/or head starts moving rapidly from side to side while the dog stands^48^
Bark	Head and lips forward, mouth opening and shutting repeatedly to omit a large, sharp, short sound emitted from the throat^38^
Howl	Raised muzzle perpendicular to ground and emits a long, drawn-out sound through semi-closed jaws. Stops when dog lowers head and/or when sound is no longer produced (*modified*)^38^

Additionally, three trained dog behaviour coders provided QBA scores on all videos collected during this study, using a list of terms (Table 3.) modified from previous research assessing dog emotional states in different settings^22,49^. New terms (‘agitated’, ‘calm’, ‘confident’ and ‘happy’) were added to ensure inclusion of terms covering a range of emotional states from across the four emotion quadrants. After watching each 10-min video, coders provided one score per term. Terms were scored using a visual analog scale, where a score of 0 was given when the dogs were expressing a total lack of, or negligible amount, of the emotion indicated by the term, and a score of 124 was given when the dog was strongly expressing the emotion indicated by the term. A random selection of 10 videos (2.9%) were re-coded by all three coders for a total of three repetitions, with repeats being randomly distributed throughout the course of data collection, to assess intra-rater reliability. As with the video coding, the number of videos were selected based on a review of literature^42–44^ and video name was encoded in order to blind the raters to which videos were repetitions.

**Table 3:** List of QBA terms used to assess dog emotion during test sessions.

Term	Definition
Agitated	Disturbed, upset, hyperactive (*defined for this study*)
Alert	Vigilant, inquisitive, on guard^22^
Bored	Disinterested, passive, showing sub-optimal arousal levels/drowsiness signs^22^
Calm	Absence of strong positive/negative emotions (*defined for this study*)
Comfortable	Without worries, settled in environment, peaceful with external stimuli (*modified*)^22^
Confident	Self-assured, purposeful, unconcerned, composed (*defined for this study*)
Engaged	Actively focused on a specific object or task, attempts to interact with object^49^
Excited	Positively agitated in response to external stimuli, euphoric, exuberant, thrilled^22^
Explorative	Confident in exploring the environment or new stimuli, investigative^22^
Fearful	Timid, scared, timorous, doesn't approach people or moves away, shows postures typical of fear^22^
Frustrated	Annoyed, irritable, restless, unable to obtain what it wants, impatient^49^
Happy	Delighted, pleased, joyful, content (*defined for this study*)
Interested	Attentive, attracted to stimuli and attempting to approach them^22^
Nervous	Uneasy, agitated, shows fast arousal, unsettled, restless, hyperactive^22^
Relaxed	Easy going, calm or acting in a calm way, doesn't show tension^22^
Sad	Low arousal, unhappy, downcast, depressed (*modified*)^49^
Stressed	Tense, shows signs of distress^22^
Tense	Stiff, rigid posture, on edge^49^

At the same time as providing QBA scores, coders were also instructed to score the emotional valence (i.e., how emotionally positive or negative they perceived the dog to be) and arousal (i.e., the intensity they perceived the dog’s emotional state to be) of the dog. In order to allow for more granularity in the response of the coders a visual analog scale ranging from 0 to 124 was used in place of the 1 to 7 scale implemented in the pilot study. The left-hand side of the scale (score 0) was defined as a very negative emotional state, or very low arousal/calm emotional state. The right-hand side of the scale (score 124) was defined as a very positive emotional state, or very highly aroused/excited emotional state. These scores were used to confirm the dogs responded to the scenarios as anticipated but were not otherwise used in the data analysis.

#### Temperature parameters

A portable infra-red camera (FLIR T840, FLIR, OR, USA) was used to capture infra-red videos for measurement of the surface temperature of the eye and nose of the dog. The infra-red camera had a thermal range of − 20 to 150 °C and a resolution of 464 × 348 pixels. Additionally, the camera has an accuracy of ± 2 °C or ± 2% of reading and sensitivity to detect temperature differences within a frame of < 30 mK. During video recordings the value of emissivity was set at 1 as per manufacturer guidelines. Dogs were recorded in a climate-controlled hallway immediately after the end of the test session prior to entry to the post-test sampling room. The camera was positioned on a tripod approximately 1-m away from the dog, with the lens parallel to the floor and in line with the dog’s head. To minimize the effect of external temperature on infra-red readings all testing and sampling areas were maintained at 18 ± 2 °C. The temperature of the test room (or car for car test sessions), hallway, sampling room, and outside temperature were monitored and recorded at the end of every test session using digital thermohygrometers (Doqaus, Shenzhen, China).

Following infra-red video recording, dogs proceeded into the sampling room, where tympanic temperature of both the right and left ear was measured using an infra-red thermometer (Braun Thermoscan 7 IRT6520; Frankfurt, Germany) with probe covers inserted into the dog’s ear canal. The thermometer has a reported accuracy of ± 0.2 °C. The difference between left and right ear temperature was then calculated by subtracting the right ear temperature from the left ear temperature.

**Figure 3:**
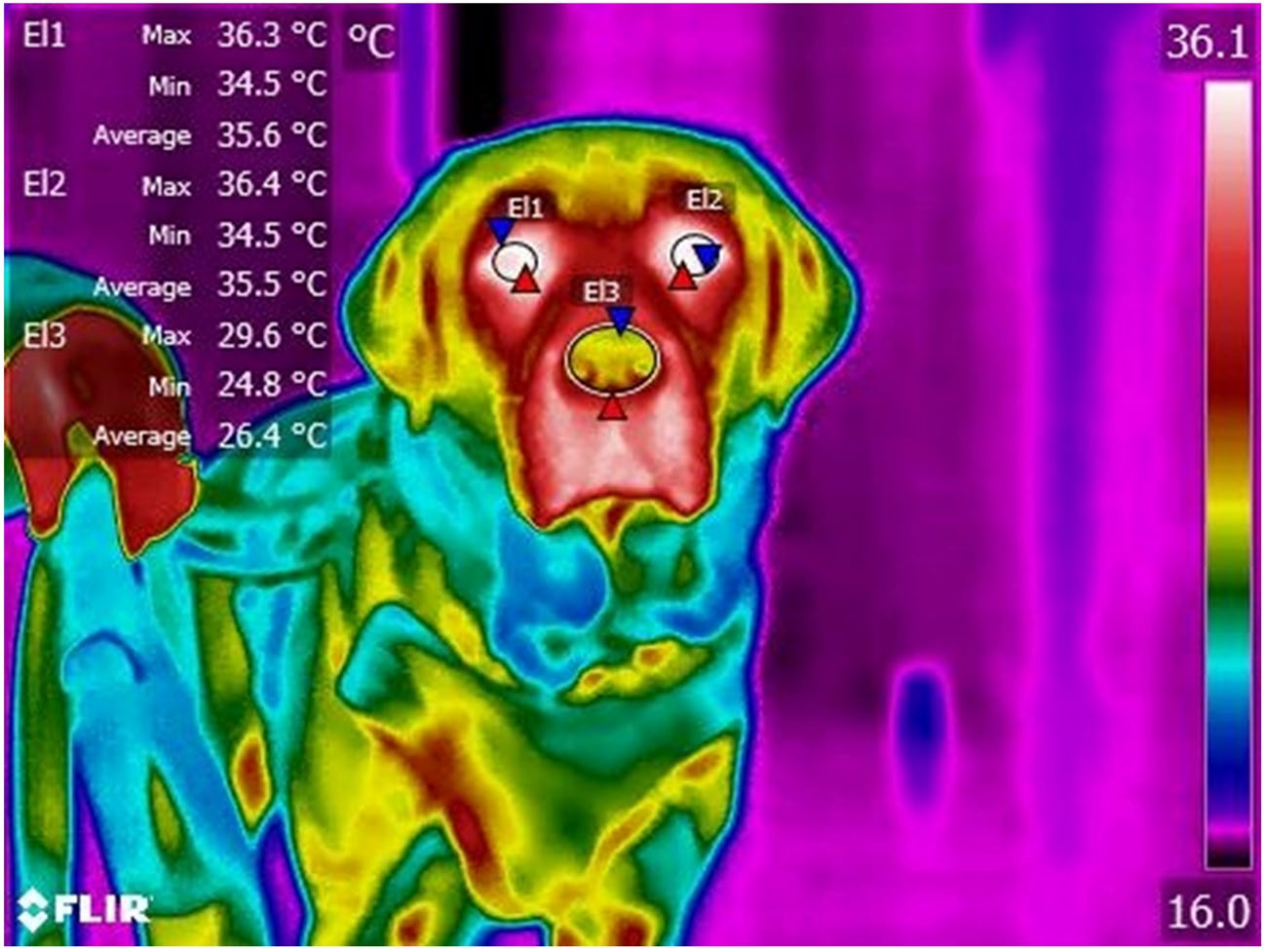
Example infra-red thermographic image indicating location of ellipses used to measure surface temperature of the eyes and nose of a dog. Image is of Labrador Retriever taken using FLIR T840 and analyzed using FLIR Tools software. E|1 shows the placement of an ellipse around the right eye. E|2 shows the placement of an ellipse around the left eye. E|3 shows the placement of an ellipse around the nose. Maximum, minimum, and mean temperatures within these ellipses are displayed as text on the image.

Video footage from the infra-red camera was analysed using FLIR Tools software (FLIR, OR, USA). The frame in which the dog directly faced the camera and was most in focus was selected for temperature capture. Mean left and right eye temperature were collected through the use of an ellipse drawn within the anterior surface region of each eye. Mean nose temperature was collected from an ellipse drawn encompassing the anterior surface of the nose (Fig. 3). The difference between left and right eye temperature was then calculated by subtracting the right eye temperature from the left eye temperature.

#### Blood parameters

Following collection of body temperature parameters, blood samples were collected in order to measure serum cortisol, serum serotonin, and plasma ACTH. Prior to sampling, a small patch of hair was shaved from the injection site on the dog’s neck. A disinfectant wipe (Vetasept; Animalcare Ltd, York, UK) and topical anesthesia (Ethycalm Plus; Invicta, West Sussex, UK) was then applied to the area before a 3.2 mL blood sample was collected from the jugular vein by a qualified technician. In order to minimize the impact of collection stress on these parameters, blood collection was terminated if not completed within five minutes of the end of the scenario.

Blood samples for cortisol and serotonin analysis were collected into serum gel tubes and left to stand for 30 min before being transported to the onsite laboratory. These samples were spun down using a centrifuge set at 2000*g* for 10 min at ambient temperature within two hours of collection before being aliquoted and stored at − 80 °C in preparation for later analyses. Blood samples for ACTH were collected into EDTA tubes, inverted 10 times and immediately stored on ice for transportation to the onsite laboratory for further processing. These samples were spun down using a centrifuge at 2000*g* for 10 min at 4 °C within an hour of collection before being aliquoted and stored at − 80 °C until analysis.

Cortisol analysis was performed in-house using the R&D Systems, Parameter Cortisol Immunoassay (bio-techne, Minneapolis, USA) following the manufacturer’s protocol with an intra-assay variation of < 10%. Serotonin and ACTH were shipped on dry ice to an external laboratory (Nationwide Specialist Laboratories, Cambridge, UK) for analysis. There, serotonin was analysed using the Enzo LifeSciences Serotonin ELISA (Enzo Life Science, Lausen, Switzerland) while ACTH was analysed using the Biomerica ACTH ELISA Kit (Biomerica, Irvine, USA). Both tests were performed in accordance with the manufacturers protocol.

#### Salivary parameter

Following blood collection, saliva samples for analysis of sIgA were collected using Salimetrics Childrens’ Saliva Swabs (Salimetrics, LLC, Carlsbad, California, USA). Twenty minutes prior to saliva collection, food was withheld from dogs, with exception of the food provided during food-based interventions, to minimize potential contamination to the sample. One end of the swab was inserted into the dog's buccal cavity, targeting the lower gum line behind the end molar where saliva pooled, and held in position for 30 s. The end of the swab was then removed and placed into the collection tube before the unused end was used to collect saliva from the other side of the dog's mouth. Both swab tips were placed within the same collection tube, which was immediately placed on ice until transported to the onsite laboratory. In order to minimize the impact of collection stress on this parameter, saliva collection was terminated if not completed within 15 min of the end of the scenario.

At the onsite laboratory, saliva swabs were spun down in a centrifuge at 4 °C sequentially at 1000*g* for 5 min, followed by 2000*g* for 5 min and finally 5000*g* for 10 min. Samples were then stored at − 80 °C until analysis. Salivary sIgA was analysed in-house using the Abcam IgA Dog ELISA Kit following the manufacturer’s protocol with an intra-assay variation of < 10%.

### Statistical analysis

All analyses were performed using R Statistical Software version 4.2.2^50^. Inter- and intra-rater reliability of the QBA scores and behavioural coding was assessed using Intraclass Correlation Coefficients (ICCs) from a two-way mixed effects model using the R package ‘irr’^51^. Consistency agreement was used for inter-rater reliability, and absolute agreement was used for intra-rater reliability^52^. These values were interpreted as poor (ICC < 0.50), moderate (ICC: 0.50–0.75), good (ICC: 0.75–0.90) or excellent (ICC > 0.90)^52^. Consistency agreement from ICCs were also used to determine the agreement between manual coding, and the multi-parameter harness for position data.

QBA terms with poor (ICC < 0.50) inter-rater reliability, or poor intra-rater reliability for multiple coders were excluded from further analyses. The remaining QBA terms were then summarized using a principal components analysis (PCA) via the ‘FactoMineR’ R package^53^. Prior to the PCA being conducted, the suitability of data for inclusion was tested using the ‘performance’ R package^54^. Data met the requirements of a Kaiser–Meyer–Olkin (KMO) measure of sampling adequacy with KMO values > 0.50 (overall KMO = 0.91) and a significant Bartlett’s test of sphericity (*p* < 0.001)^55^. When retained PCA components were interpreted, terms with loadings ≥ |0.50| were considered to be salient. Component scores were generated using each terms weighting on the key components. Inter- and intra-rater reliability of the component scores^56^ was assessed using ICCs as described above.

To understand the relationships between each collected outcome parameter and the emotional quadrants of valence and arousal, data from the scenarios without food (i.e., separation, car, petting, toy) were fitted separately to linear mixed effects models for each parameter (via ‘nlme’ R package)^57^, with the respective parameter as the response variable, valence (negative vs positive) and arousal (low vs high) as categorical fixed effects (negative valence and low arousal as the reference categories), plus the two-way interaction between valence and arousal, and animal nested within breed as the random effects structure (intercept-only). Variance weights by arousal level were also incorporated into the models to compensate for heteroscedasticity between high and low arousal scenarios. Outdoor temperature was included as an additional (continuous) fixed effect within models exploring temperature parameters (with the exception of models pertaining to lateralised differences in temperature). Model residuals were plotted and assessed by visual inspection, and parameters were log-transformed if judged to violate model assumptions. The estimated means (back-transformed where appropriate) and 95% confidence intervals (95% CI) were extracted from the model and plotted via the R package ‘ggplot2’^58^. The significance of the fixed effects were assessed using Wald’s test via the R package ‘car’^59^. Pairwise planned comparisons were also performed, between valence levels within each arousal category, between arousal levels within each valence category, and for the two-way interaction (i.e., the difference in the differences), and multiplicity adjusted *p*-values reported. Family-wise error rate (FWER) adjustment was made using the ‘single-step’ approach of the R package ‘multcomp’ (according to the multivariate t distribution)^60^, to control for α-inflation across comparisons within each model. Further, a Bonferroni adjusted α criterion for significance of α = 0.01 was used, based on analysis of five primary parameters (i.e., Cortisol, HR, HRV-RMSSD, QBA PC1_Valence, QBA PC2_Arousal). Secondary analyses of additional parameters applied the same α criterion, to maintain a consistent Type 1 error rate across all measures.

Infrequent behaviours of shake and whining (occurring in < 50% of observations) were analyzed as present/absent for occurrence using binomial generalized linear mixed-effects models (via ‘lme4’ R package)^61^, using the same model and pairwise contrast structure as specified above, with some variations. First, excluding variance weighting, which is inappropriate for binary logistic models. Second, due to absence of whining behaviours in the positive valence conditions, this parameter was analyzed within the negative valence conditions only, with the sole categorical fixed effect of arousal and corresponding pairwise contrast between levels low/high. The estimated probabilities of the dogs performing the behaviour and 95% CIs were extracted from the model and plotted. The behaviours of barking, yawning, and howling were not analyzed due to rare occurrence (< 10% of observations).

To understand the influence of food on the collected parameters, and how this may interact with arousal, data from the four scenarios anticipated to elicit positive emotional states (i.e., petting, toy, chew, treat) were fit to further mixed effects models. The same model and pairwise contrast structure as defined above for assessment of emotional quadrants was used, with food (absent vs present) replacing valence as a categorical factor in the design.

## Results

### Response to scenarios

Inter-rater reliability was good for the valence ratings (ICC = 0.794) and moderate for the arousal ratings (ICC = 0.697) across the three trained raters. Intra-rater reliability for the valence ratings was good for rater 1 (ICC = 0.828) and excellent for rater 2 (ICC = 0.948) and rater 3 (ICC = 0.948). Intra-rater reliability for the arousal ratings was moderate for rater 1 (ICC = 0.561), rater 2 (ICC = 0.622), and rater 3 (ICC = 0.662).

**Figure 4:**
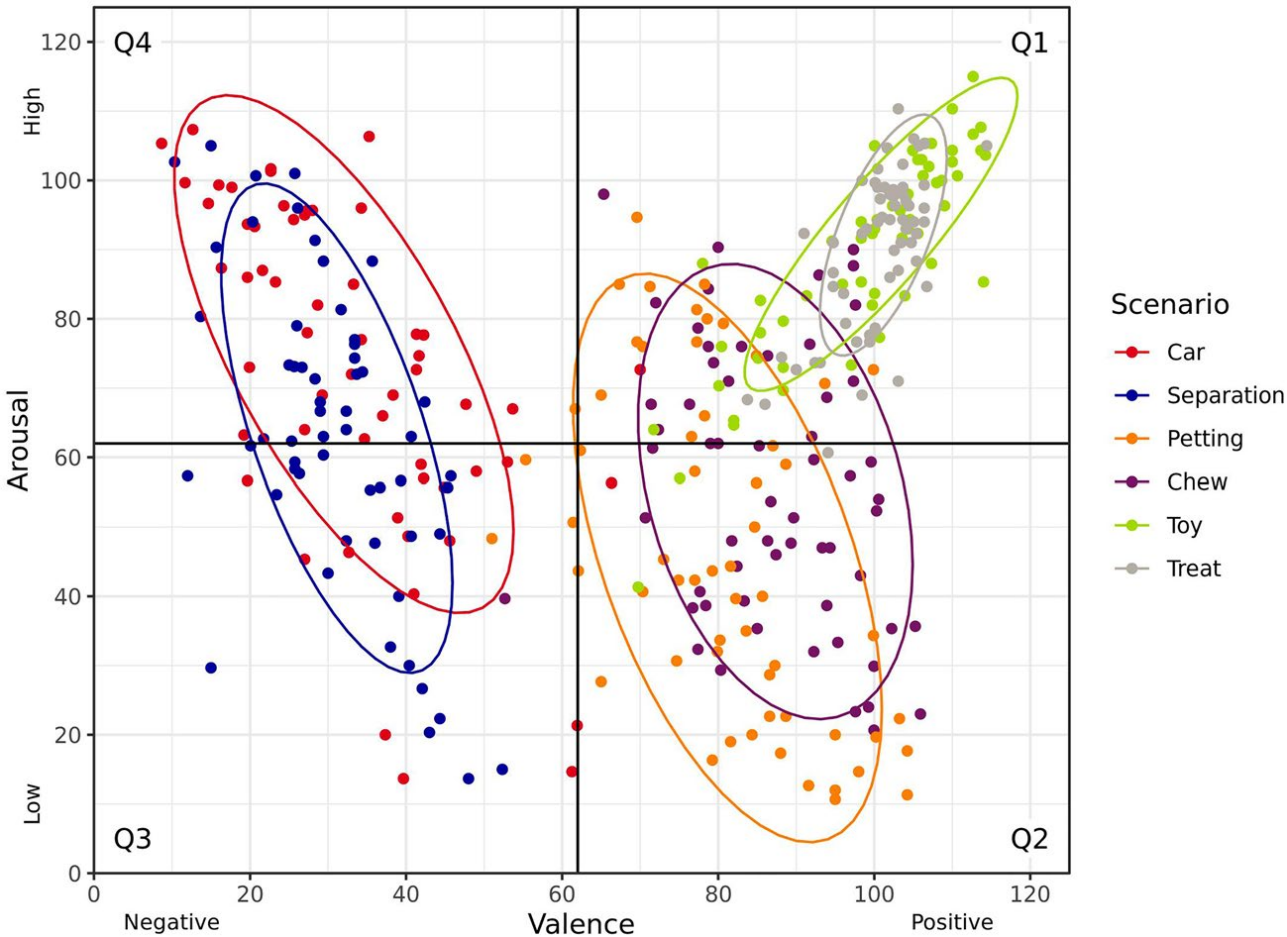
Dogs’ responses to different scenarios anticipated to elicit emotions varying from positive to negative valence and low to high arousal during the main study. Individual dog responses are indicated by mean valence and arousal ratings provided by three trained dog behaviour coders, with 80% confidence (data) ellipses for each scenario. Valence was scored on a scale from 0 (very negative) to 124 (very positive), and arousal scored from 0 (very low) to 124 (very high), with the midpoint (62) indicated.

A majority of the dogs responded as anticipated to the selected scenarios (Fig. 4). Responses were coded as being within Q1 (positive valence, high arousal) for 96.6% of dogs when exposed to the toy play scenario, and for 98.3% of dogs when exposed to the treat throwing scenario. Responses to the low arousal/positive valence scenarios were more variable, with responses coded as being with Q2 (positive valence, low arousal) for 63.8% of dogs when exposed to the petting scenario and for 62.7% of dogs when provided with a long-lasting chew. Responses were coded as being within Q4 (negative valence, high arousal) for 67.2% of dogs when exposed to the car scenario, while 45.6% of dogs were coded as being within Q3 (negative valence, low arousal) after exposure to the separation scenario.

### Primary parameters

Due to heteroscedasticity present in the residuals a log_10_-transformation was applied to models exploring cortisol and HRV-RMSSD. The other three primary parameters met model assumptions and proceeded without transformation. Estimated means for different levels of valence and arousal generated from these models are presented in Supplementary Table S1.

#### Blood serum cortisol

**Figure 5:**
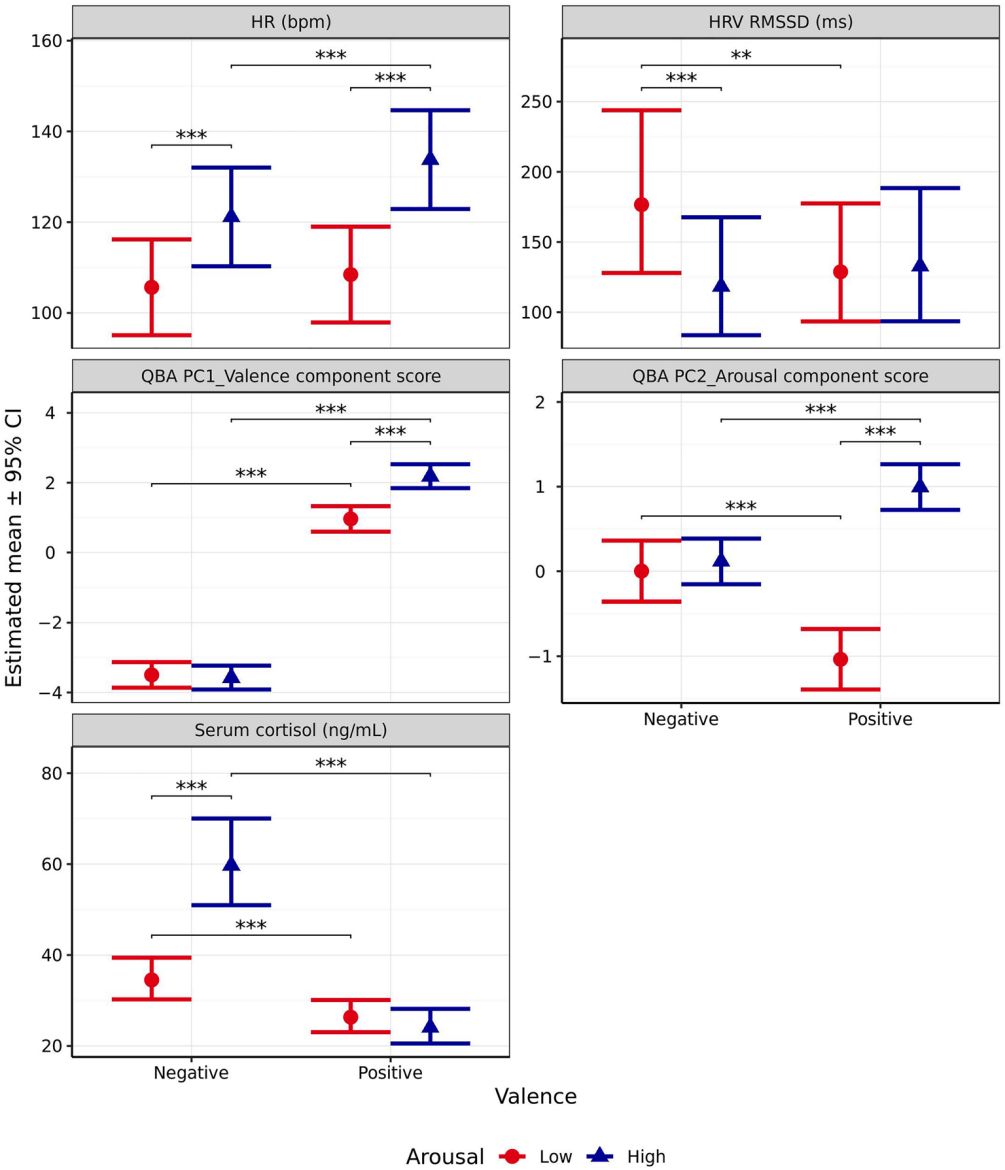
Estimated means (± 95% CI) for heart rate (bpm), heart rate variability measured using RMSSD (ms), PC1_Valence, PC2_Arousal, and blood serum cortisol (ng/mL) following test scenarios anticipated to produce responses varying in valence (negative vs positive) and arousal (high vs low). These scenarios included those anticipated to produce negative valence low arousal (separation), negative valence high arousal (car), positive valence low arousal (petting), and positive valence high arousal (toy play). ** indicates a significant difference of *p* < 0.010. *** indicates a significant difference of *p* < 0.001.

Blood serum cortisol was significantly influenced by valence (*χ*^2^_1_ = 161.1,* p* < 0.001), arousal (*χ*^2^_1_ = 33.16, *p* < 0.001), and a significant interaction effect was observed between them (*χ*^2^_1_ = 64.32 p < 0.001, Fig. 5). In scenarios designed to induce positive valence, arousal did not significantly impact cortisol levels (*p* = 0.321). However, blood cortisol was significantly higher for the high versus low arousal scenario for those designed to induce negative valence (*p* < 0.001). Blood cortisol was significantly higher for the scenarios in which dogs were anticipated to experience negative valence emotions compared to positive ones in both the low arousal (*p* < 0.001) and high arousal (*p* < 0.001) scenarios, while the interaction contrast indicated that this effect was significantly more pronounced when arousal was high (*p* < 0.001).

#### Heart rate (HR)

HR was significantly influenced by valence (*χ*^2^_1_ = 16.44, *p* < 0.001) and arousal (*χ*^2^_1_ = 156, *p* < 0.001), and a significant interaction effect was observed between them (*χ*^2^_1_ = 9.073, *p* = 0.003, Fig. 5). As expected, HR was significantly higher in scenarios designed to induce high arousal, for both the positive (*p* < 0.001) and negative (*p* < 0.001) valence scenarios. For scenarios designed to induce low arousal, valence did not significantly impact HR (*p* = 0.433), however for those designed to induce high arousal, HR was observed to be significantly higher following the scenario anticipated to induce positive valence compared to the one predicted to induce negative valence (*p* < 0.001).

#### Heart rate variability (HRV) RMSSD

Heart rate variability measured using RMSSD was only significantly impacted by the interaction between arousal and valence (*χ*^2^_1_ = 8.636, *p* = 0.003, Fig. 5), when comparing against the adjusted α criterion of 0.01. In scenarios designed to induce high arousal, no significant difference in HRV-RMSSD was observed between those anticipated to induce positive versus negative valence (*p* = 0.727). However, in scenarios designed to induce low arousal, HRV-RMSSD was significantly higher during the negative valence scenario than the positive one (*p* = 0.001). In scenarios designed to induce negative valence, HRV-RMSSD was significantly higher in the low arousal scenario than the high arousal scenario (*p* = 0.001). However, there was no significant effect of anticipated arousal between positive valence scenarios (*p* = 0.989).

#### Qualitative behavioural assessment (QBA)

Inter-rater reliability analysis demonstrated that agreement was generally moderate (ICC = 0.50–0.75) for a majority of the QBA terms, with alert, bored, explorative, fearful, and frustrated having poor agreement (ICC < 0.50) and confident and happy having good agreement (ICC = 0.75–0.90). Intra-rater reliability was variable, with agreement ranging from poor to excellent agreement depending on the term and coder (Table 4). Based on these reliability results, the terms ‘alert’, ‘bored’, ‘explorative’, ‘fearful’, and ‘frustrated’ were not included in further analyses.

**Table 4:** Intraclass correlation coefficients indicating levels of agreement both between coders (inter-rater reliability) and within each of the three coders (intra-rater reliability) for each term of the QBA. Values indicating poor reliability (ICC<0.50) are in bold.

	Inter-	Intra-		
		Rater 1	Rater 2	Rater 3
Agitated	0.567	0.518	0.888	0.831
Alert	**0.379**	0.554	0.697	0.951
Bored	**0.305**	0.802	**0.444**	**0.148**
Calm	0.619	0.615	0.932	0.928
Comfortable	0.727	0.766	0.975	0.991
Confident	0.753	0.830	0.980	0.984
Engaged	0.713	0.731	0.996	0.996
Excited	0.723	0.677	0.941	0.836
Explorative	**0.360**	0.682	0.727	**0.076**
Fearful	**0.401**	0.860	0.926	**0.424**
Frustrated	**0.389**	0.544	0.812	0.724
Happy	0.756	0.809	0.931	0.989
Interested	0.676	0.623	0.907	0.989
Nervous	0.686	0.664	0.932	0.675
Relaxed	0.631	**0.489**	0.906	0.978
Sad	0.529	0.722	0.961	0.769
Stressed	0.723	0.651	0.920	0.880
Tense	0.724	0.796	0.947	0.946

**Figure 6:**
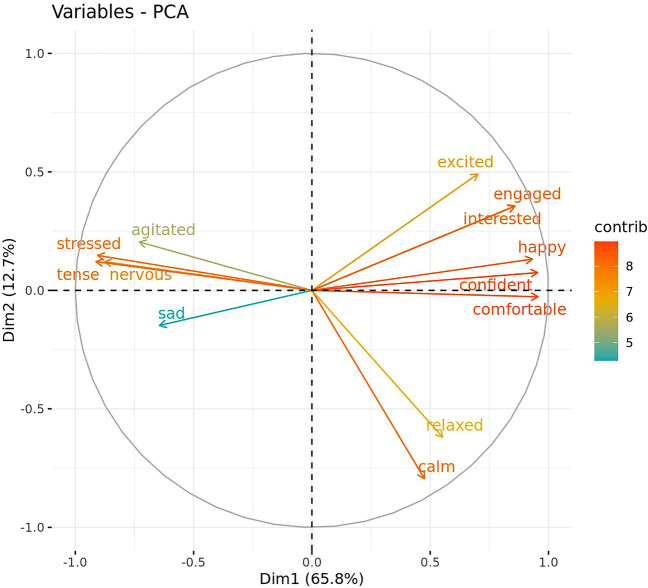
Component loadings generated from a PCA of QBA terms showing the two main components of interest (Dim1: PC1_Valence, Dim2: PC2_Arousal). Arrow color is indicative of the strength of contribution of the term.

Analysis of the QBA data using a PCA suggested two main components of interest based on the strength of loadings and the variance explained (Table 5; Fig. 6). The first component explained 65.8% of the total variance and was labelled ‘PC1_Valence’. It was comprised of positive loadings for the terms ‘comfortable’, ‘confident’, ‘happy’, ‘engaged’, ‘interested’, and ‘excited’, and negative loadings for the terms ‘tense’, ‘stressed’, ‘nervous’, ‘agitated’, and ‘sad’. The second component explained 12.7% of the total variance and was labelled ‘PC2_Arousal’. It was comprised of negative loadings for the terms ‘calm’, and ‘relaxed’. While they did not meet the a priori cut-off of ≥ |0.50|, it is worth noting that PC2_Arousal also comprised of moderate positive loadings for ‘excited’, ‘interested’, and ‘engaged’. Inter-rater reliability was good for the PC1_Valence component score (ICC = 0.840) and moderate for the PC2_Arousal component score (ICC = 0.671). Intra-rater reliability was good to excellent for all raters (ICC = 0.759–0.986) for the PC1_Valence component score but was poor for rater 1 (ICC = 0.496), good for rater 3 (ICC = 0.760) and excellent for rater 2 (ICC = 0.928) for the PC2_Arousal component score.

**Table 5:** Components extracted by the PCA of QBA scores. Loadings ≥ |0.50| are in bold.

Term	PC1_Valence	PC2_Arousal
Comfortable	**0.956**	− 0.027
Confident	**0.953**	0.075
Happy	**0.932**	0.132
Engaged	**0.859**	0.356
Interested	**0.857**	0.355
Excited	**0.702**	0.491
Tense	− **0.912**	0.121
Stressed	− **0.906**	0.147
Nervous	− **0.876**	0.121
Agitated	− **0.729**	0.204
Sad	− **0.644**	− 0.148
Calm	0.475	− **0.795**
Relaxed	0.552	− **0.619**

Based on the reliability results, scores from rater 1 were excluded from analysis of PC2_Arousal. In order to minimize the impact of inter-rater variation, an average score from all three coders was used for analysis of PC1_Valence, and from rater 2 and 3 for analysis of PC2_Arousal. With these modifications, the QBA component scores were considered sufficiently reliable for further analysis.

Unsurprisingly, PC1_Valence was significantly affected by the anticipated valence of a scenario (*χ*^2^_1_ = 1688, *p* < 0.001), with higher values being observed when valence was anticipated to be positive. Interestingly, PC1_Valence was also significant impacted by anticipated arousal (*χ*^2^_1_ = 20.92, *p* < 0.001) and the interaction effect between arousal and valence (*χ*^2^_1_ = 26.58, *p* < 0.001, Fig. 5). PC1_Valence was significantly higher for scenarios predicted to induce positive valence regardless of induced arousal level (both *p* < 0.001). When valence was anticipated to be negative, there was no significant difference in PC1_Valence between the high and low arousal scenarios (*p* = 0.966). However, for scenarios anticipated to induce positive valence, PC1_Valence was significantly higher for the high versus low arousal scenario (*p* < 0.001). The interaction contrast further indicated that the difference in these differences was itself significant (*p* < 0.001).

Similarly, PC2_Arousal was significantly affected overall by both the predicted arousal (*χ*^2^_1_ = 112, *p* < 0.001) and valence of a scenario (*χ*^2^_1_ = 14.32*, p* < 0.001), as well as the interaction between the two (*χ*^2^_1_ = 88.36, *p* < 0.001, Fig. 5). However, pairwise comparisons revealed that for scenarios anticipated to induce negative valence there was no significant difference in PC2_Arousal between those designed to produce high and low arousal (*p* = 0.829). By contrast, when valence was predicted to be positive, PC2_Arousal was significantly higher for the scenario anticipated to induce high, rather than low levels of arousal (*p* < 0.001). Interestingly, for low arousal scenarios, PC2_Arousal was significantly higher when valence was anticipated to be negative compared to positive (*p* < 0.001), whereas, when arousal was predicted to be high, the inverse relationship was found (*p* < 0.001). Further, the interaction indicated that this difference in differences was itself significant (*p* < 0.001).

### Secondary parameters

Due to heteroscedasticity present in the residuals, a log-transformation was applied to the models for serotonin, ACTH, sIgA, HRV-SDRR, and panting. All remaining parameters met model assumptions and proceeded without transformation. Estimated means and probabilities for different levels of valence and arousal generated from these models are presented in Supplementary Table S1.

#### Blood serum serotonin

**Figure 7:**
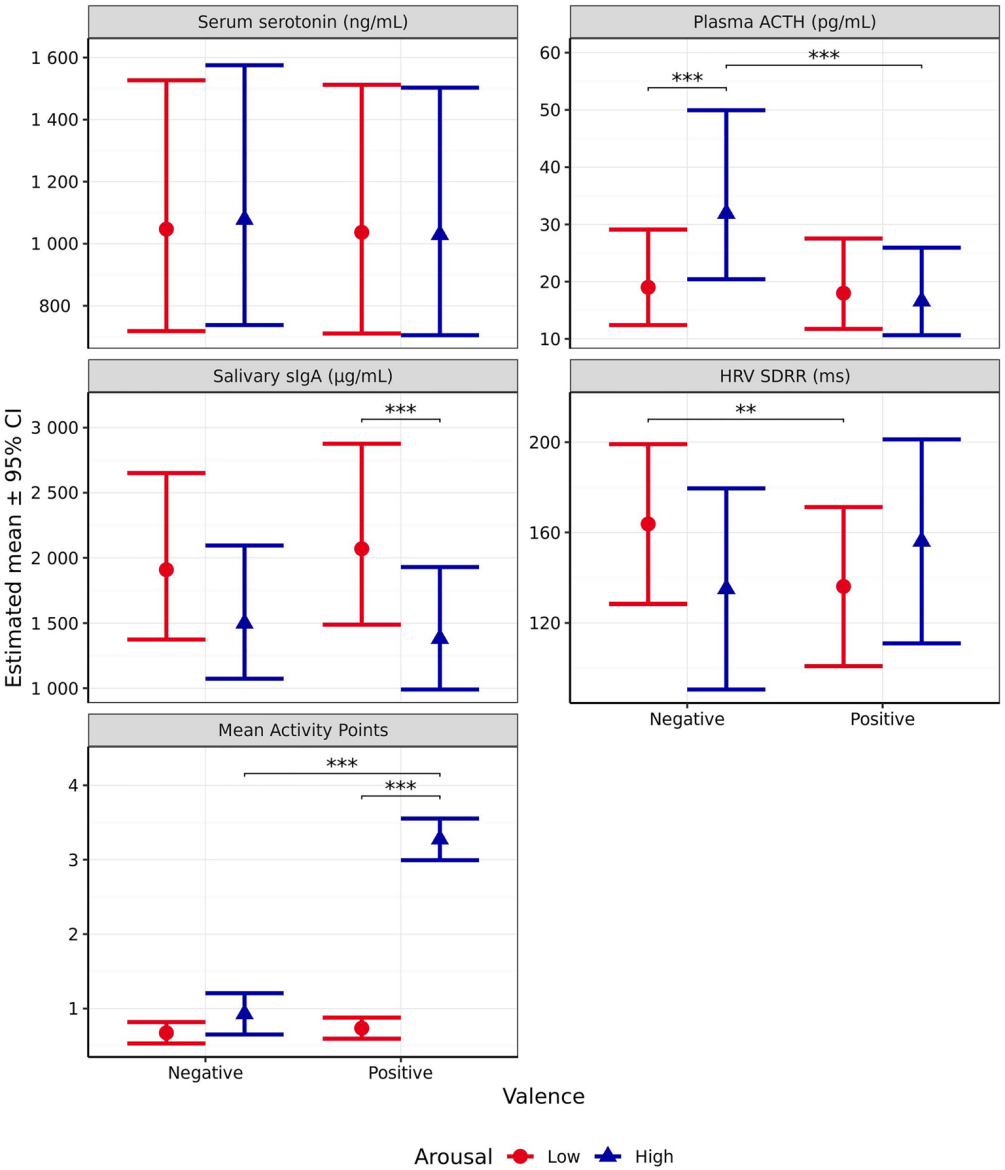
Estimated means (± 95% CI) for blood serum serotonin (ng/mL), plasma ACTH (pg/mL), salivary sIgA (µg/mL), heart rate variability measured using SDRR (ms), and mean Activity Points following test scenarios anticipated to produce responses varying in valence (negative vs positive) and arousal (high vs low). These scenarios included those anticipated to produce negative valence low arousal (separation), negative valence high arousal (car), positive valence low arousal (petting), and positive valence high arousal (toy play). *** indicates a significant difference of *p* < 0.001.

Blood serum serotonin level was not significantly affected by the anticipated emotional quadrant with regards to arousal (*χ*^2^_1_ = 0.131, *p* = 0.718), valence (*χ*^2^_1_ = 0.769, *p* = 0.381), or the interaction between the two (*χ*^2^_1_ = 0.373, *p* = 0.541, Fig. 7).

#### Blood plasma ACTH

Blood plasma ACTH was significantly impacted by both valence (*χ*^2^_1_ = 15.67, *p* < 0.001) and arousal (*χ*^2^_1_ = 12.92, *p* < 0.001), with a significant interaction between the two (*χ*^2^_1_ = 24.63, *p* < 0.001, Fig. 7). When valence was anticipated to be positive, arousal did not significantly impact ACTH (*p* = 0.744). However, when valence was anticipated to be negative, ACTH was significantly higher for the high arousal scenario than the low arousal scenario (*p* < 0.001). For scenarios expected to induce low arousal, ACTH levels did not significantly differ between those anticipated to induce positive and negative valences (*p* = 0.754). However, for scenarios anticipated to induce high arousal, ACTH levels were significantly higher following the negative versus positive valence scenario (*p* < 0.001). In addition, the interaction contrast indicated that this difference in differences was itself significant (*p* < 0.001).

#### Salivary sIgA

Salivary sIgA was significantly affected by anticipated arousal (*χ*^2^_1_ = 28.35, p < 0.001, Fig. 7) but not valence (*χ*^2^_1_ = 0.016, *p* = 0.898), with no significant interaction between the two (*χ*^2^_1_ = 1.783, *p* = 0.182). Salivary sIgA was significantly higher following the low versus high arousal scenario for those anticipated to induce positive valence (*p* < 0.001) but non-significantly so for scenarios designed to induce negative valence, when comparing against the adjusted α criterion of 0.01 (*p* = 0.020). However, the interaction contrast, representing the difference in these differences, was also non-significant (*p* = 0.485).

#### Heart rate variability (HRV) SDRR

Heart rate variability measured using SDRR was not significantly impacted by valence (*χ*^2^_1_ = 5.375, *p* = 0.020), arousal (*χ*^2^_1_ = 0.211, *p* = 0.646), or the interaction between valence and arousal (*χ*^2^_1_ = 5.559, *p* = 0.018, Fig. 7), when comparing against the adjusted α criterion of 0.01. Looking at the individual pairwise comparisons, in scenarios designed to induce high arousal, no significant difference in HRV-SDRR was observed between those anticipated to induce positive versus negative valence (*p* = 0.622). However, in scenarios designed to induce low arousal, HRV-SDRR was significantly higher during the negative valence scenario than the positive one (*p* = 0.008). There was no significant effect of arousal on HRV-SDRR within either the positive valence scenarios (p = 0.470) or the negative valence scenarios (p = 0.161). Further, the interaction contrast was also non-significant (p = 0.067).

#### Mean activity points

Anticipated arousal (*χ*^2^_1_ = 267.9, *p* < 0.001), valence (*χ*^2^_1_ = 52.15, *p* < 0.001), and the interaction between the two (*χ*^2^_1_ = 180.5, *p* < 0.001, Fig. 7) were all observed to significantly influence mean Activity Points. When arousal was anticipated to be low, mean Activity Points did not significantly differ with regards to scenario valence (*p* = 0.790). However, when arousal was anticipated to be high, the scenario predicted to induce positive valence resulted in a significantly higher mean Activity Scores than the scenario predicted to induce negative valence (*p* < 0.001). The high arousal scenario resulted in significantly higher mean Activity Points than the low arousal scenario when valence was anticipated to be positive (*p* < 0.001), whereas this was not the case for scenarios predicted to induce negative valence (*p* = 0.118). Further, the interaction contrast indicated that this difference in differences was itself significant (*p* < 0.001).

#### Mean eye temperature

**Figure 8:**
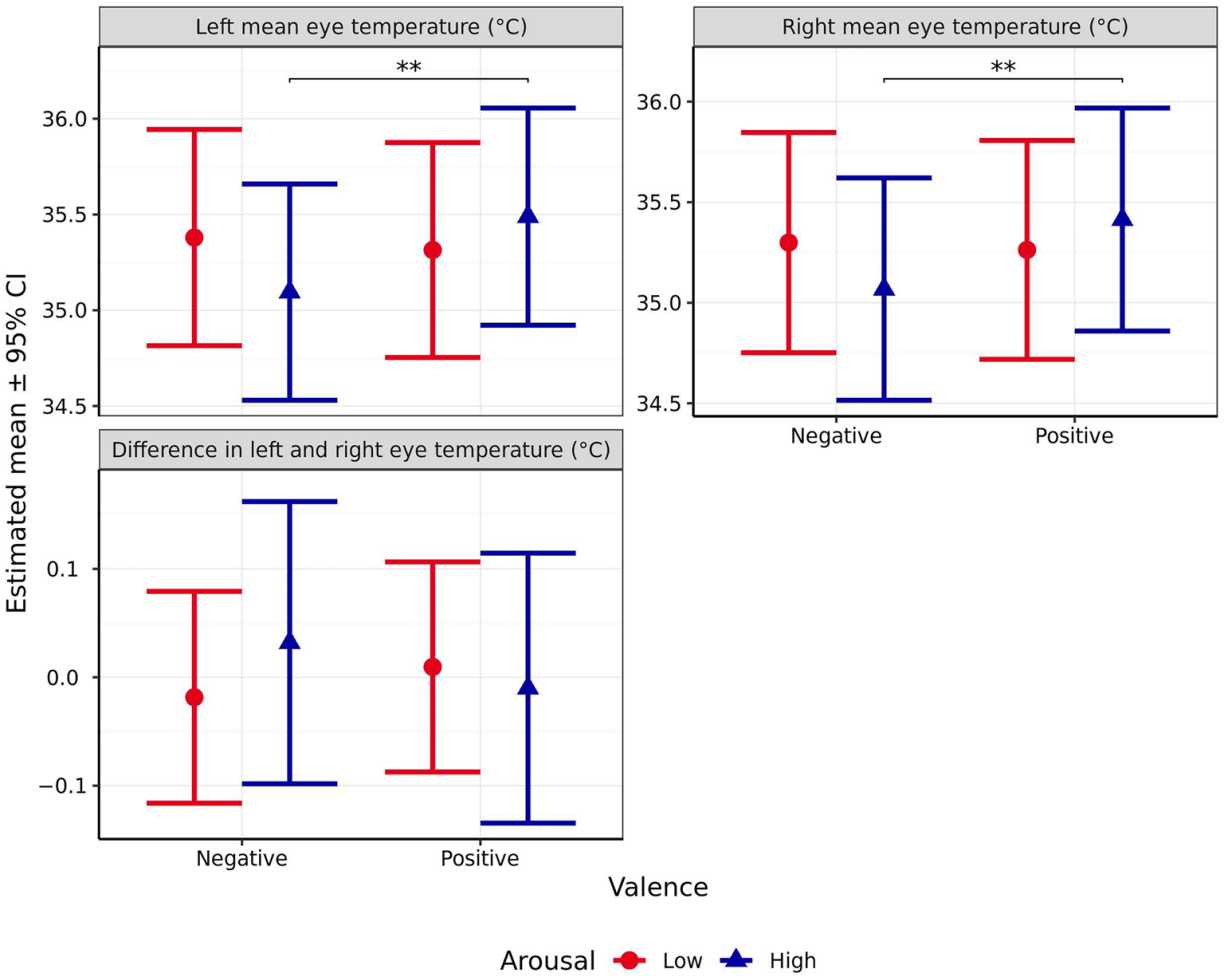
Estimated means (± 95% CI) for mean left eye temperature, mean right eye temperature, and the difference in left and right eye temperatures (all °C) following test scenarios anticipated to produce responses varying in valence (negative vs positive) and arousal (high vs low). These scenarios included those anticipated to produce negative valence low arousal (separation), negative valence high arousal (car), positive valence low arousal (petting), and positive valence high arousal (toy play). ** indicates a significant difference of *p* < 0.010.

Outside temperature significantly affected right mean eye temperature (*χ*^2^_1_ = 10, *p* = 0.002) but not left mean eye temperature (*χ*^2^_1_ = 4.826, *p* = 0.028). Furthermore, while no significant effect of arousal (Left: *χ*^2^_1_ = 0.428, *p* = 0.513, Right:* χ*^2^_1_ = 0.238, *p* = 0.626) or valence was observed (Left: *χ*^2^_1_ = 4.12, *p* = 0.042, Right:* χ*^2^_1_ = 3.033, *p* = 0.082), mean eye temperature was significantly influenced by the interaction between the two (Left: *χ*^2^_1_ = 9.931, *p* = 0.002, Right:* χ*^2^_1_ = 7.651, *p* = 0.006*,* Fig. 8). When arousal was anticipated to be high, mean eye temperature was significantly higher following the positive compared to the negative valence scenario (Left: *p* = 0.001 Right: *p* = 0.005). However, no significant difference was observed when arousal was anticipated to be low (Left: *p* = 0.893 Right: *p* = 0.970). Similarly, there were no significant differences in mean eye temperature between the high and low arousal scenario, for either eye, regardless of valence (all p > 0.01). In addition, for the left eye only, the difference in differences was found to be significant (Left: *p* = 0.007 Right: *p* = 0.023).

No significant effect of arousal (*χ*^2^_1_ = 0.114 *p* = 0.736), valence (*χ*^2^_1_ = 0.009, *p* = 0.923), or their interaction (*χ*^2^_1_ = 0.694, *p* = 0.405, Fig. 8) was observed on the difference in mean eye temperature between the left and right eye.

#### Ear temperature

**Figure 9:**
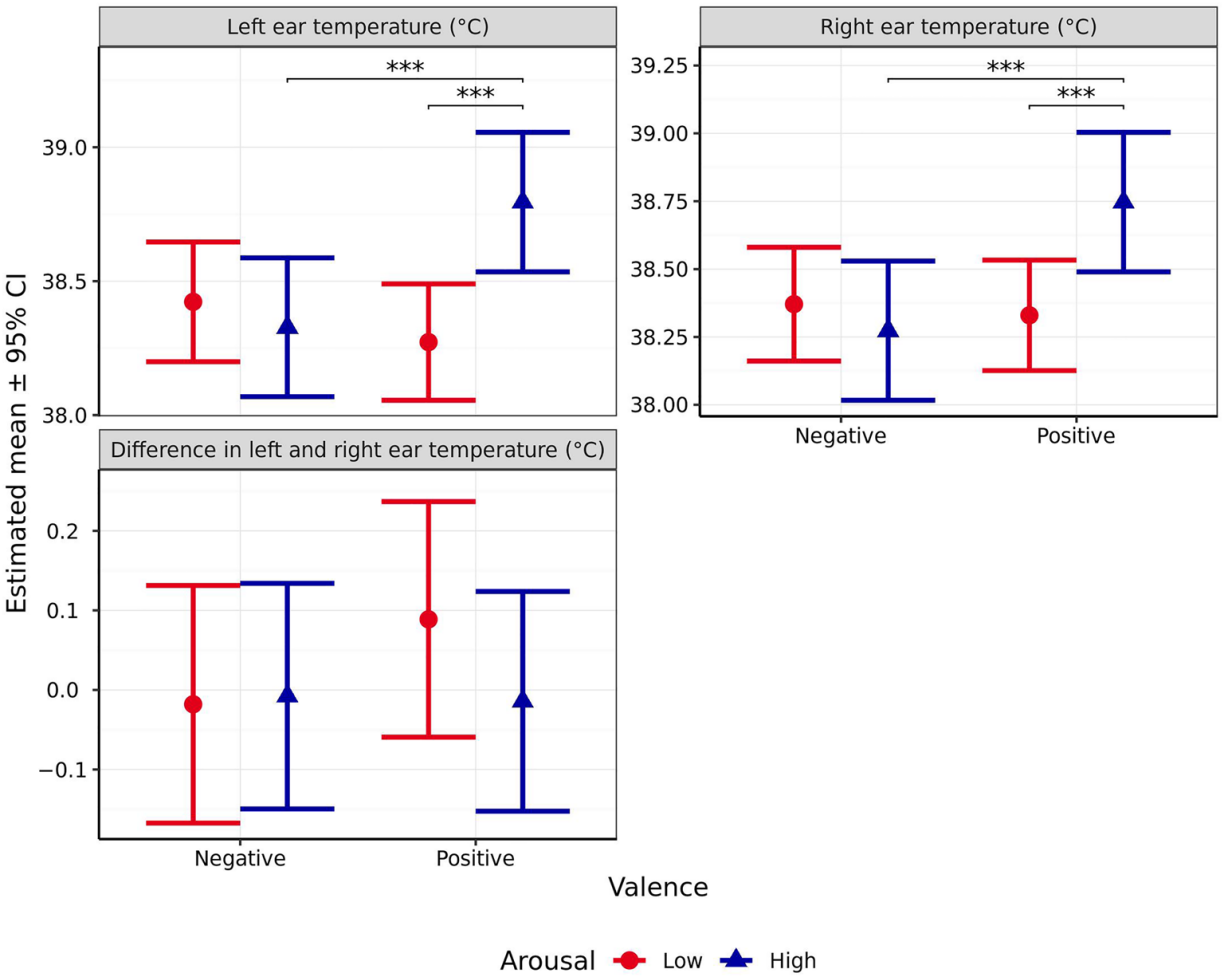
Estimated means (± 95% CI) for mean left ear temperature, mean right ear temperature, and the difference in left and right ear temperatures (all °C) following test scenarios anticipated to produce responses varying in valence (negative vs positive) and arousal (high vs low). These scenarios included those anticipated to produce negative valence low arousal (separation), negative valence high arousal (car), positive valence low arousal (petting), and positive valence high arousal (toy play). *** indicates a significant difference of *p* < 0.001.

Left and right ear temperature were not significantly affected by outside temperature (Left: *χ*^2^_1_ = 2.895, *p* = 0.089; Right: *χ*^2^_1_ = 5.269, *p* = 0.022) or anticipated valence (Left: *χ*^2^_1_ = 0.526, *p* = 0.468; Right: *χ*^2^_1_ = 2.814, *p* = 0.093). However, significant effects of arousal (Left: *χ*^2^_1_ = 12.9, *p* < 0.001; Right: *χ*^2^_1_ = 7.395, *p* = 0.007), and the interaction between the two emotional dimensions (Left: *χ*^2^_1_ = 26.39, *p* < 0.001; Right: *χ*^2^_1_ = 17.92, *p* < 0.001, Fig. 9) were observed. When valence was anticipated to be positive, ear temperature was significantly higher when the scenario was predicted to induce high arousal (Left: *p* < 0.001, Right: *p* < 0.001). However, no effect of arousal was observed when valence was anticipated to be negative (Left: *p* = 0.632, Right: *p* = 0.624). For high arousal scenarios, ear temperature was significantly higher when valence was anticipated to be positive compared to when it was anticipated to be negative (Left: *p* < 0.001, Right: *p* < 0.001). However, for low arousal scenarios, no significant difference was observed between valence levels (Left: *p* = 0.088, Right: *p* = 0.886). Further, interaction contrasts indicated that these differences in differences were themselves significant, for both ears (Left: *p* < 0.001, Right: *p* < 0.001).

Lateralised difference in ear temperature (i.e., between the left and right ears) was not significantly impacted by valence (*χ*^2^_1_ = 0.72, *p* = 0.396), arousal (*χ*^2^_1_ = 0.755, *p* = 0.385), or the interaction between valence and arousal (*χ*^2^_1_ = 1.076, *p* = 0.299, Fig. 9).

#### Mean nose temperature

**Figure 10:**
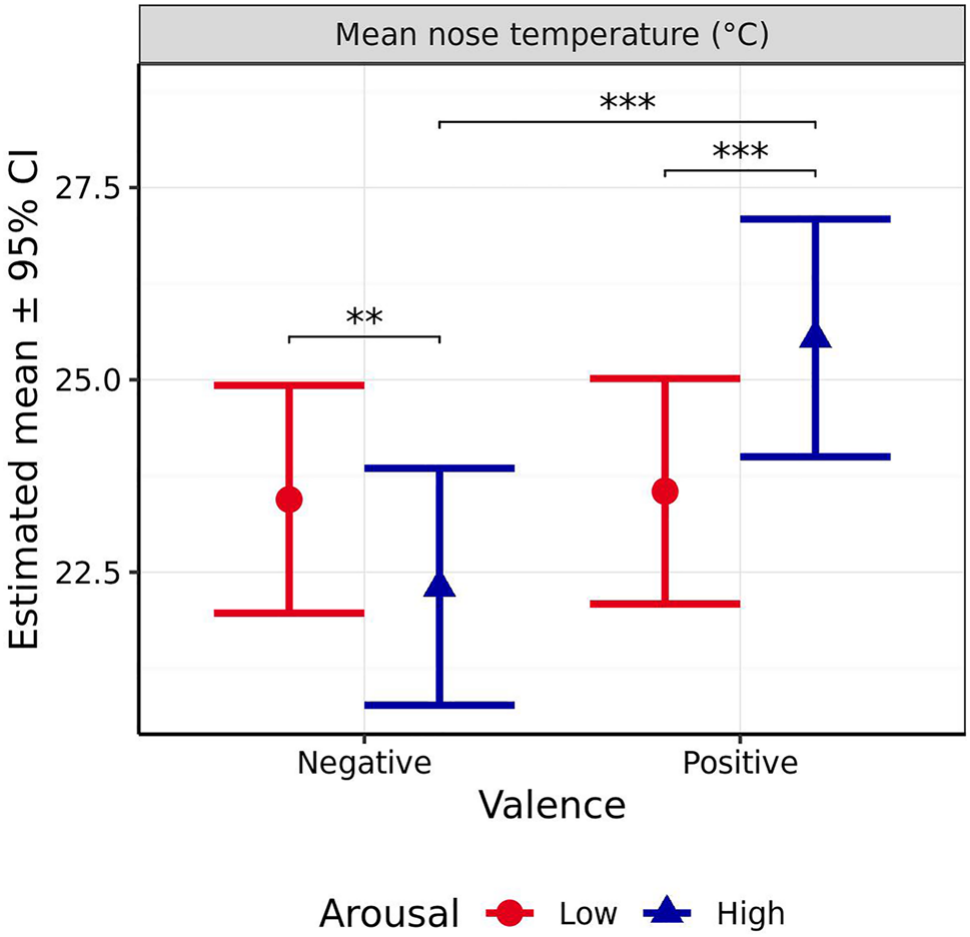
Estimated means (± 95% CI) for mean nose temperature (°C) following test scenarios anticipated to produce responses varying in valence (negative vs positive) and arousal (high vs low). These scenarios included those anticipated to produce negative valence low arousal (separation), negative valence high arousal (car), positive valence low arousal (petting), and positive valence high arousal (toy play). ** indicates a significant difference of *p* < 0.010. *** indicates a significant difference of *p* < 0.001.

Mean nose temperature was significantly affected by outside temperature (*χ*^2^_1_ = 79.89, *p* < 0.001), anticipated scenario valence (*χ*^2^_1_ = 23.35, *p* < 0.001), and the interaction between anticipated valence and arousal (*χ*^2^_1_ = 38.16, *p* < 0.001, Fig. 10), but not by anticipated arousal (*χ*^2^_1_ = 3.684, *p* = 0.055). When valence was anticipated to be negative, mean nose temperature was significantly higher after the low versus the high arousal scenario (*p* = 0.008), while the opposite effect was observed when valence was anticipated to be positive (*p* < 0.001). When arousal was anticipated to be high, the positive valence scenario preceded significantly higher mean nose temperatures than the negative valence scenario (*p* < 0.001), whereas when arousal was expected to be low, there was no significant effect of scenario valence on nose temperature (*p* = 0.981). The interaction contrast indicated that this difference in differences was itself significant (*p* < 0.001).

#### Behavioural parameters

Results of agreement analysis for proportion of time in different positions measured using manual coding and the multi-parameter harness indicated agreement was moderate for sitting (ICC = 0.732), standing (ICC = 0.667) and lying (ICC = 0.629). The use of a multi-parameter harness to measure position was therefore deemed acceptable for analysis. Yawning, barking, and howling occurred infrequently and therefore were not included in further analyses. Intra-rater reliability was excellent (ICC > 0.90) for the remaining behaviours.

**Figure 11:**
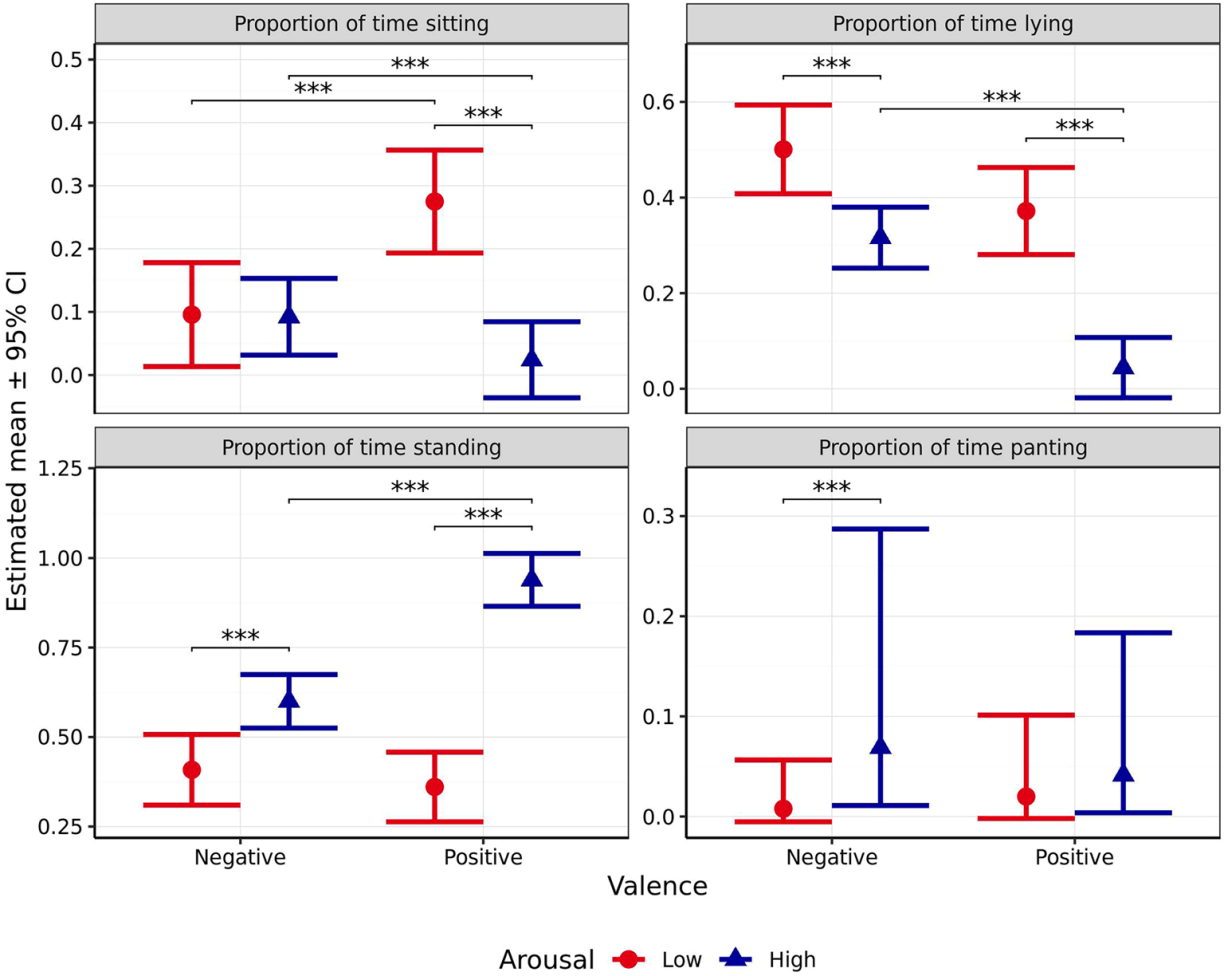
Estimated means (± 95% CI) for proportion of time spent sitting, lying, standing, and panting during test scenarios anticipated to produce responses varying in valence (negative vs positive) and arousal (high vs low). These scenarios included those anticipated to produce negative valence low arousal (separation), negative valence high arousal (car), positive valence low arousal (petting), and positive valence high arousal (toy play). *** indicates a significant difference of *p* < 0.001.

Proportion of time spent sitting was significantly affected by arousal (*χ*^2^_1_ = 41.09, *p* < 0.001) and the interaction between arousal and valence (*χ*^2^_1_ = 37.56, *p* < 0.001), but not valence alone (*χ*^2^_1_ = 2.361, *p* = 0.124, Fig. 11). Time spent sitting did not significantly differ between scenarios predicted to induce high and low arousal when valence was anticipated to be negative (*p* = 0.999), although when valence was anticipated to be positive, dogs spent significantly less time sitting during the high arousal scenario than the low arousal scenario (*p* < 0.001). When arousal was predicted to be low, the negative valence scenario induced significantly less sitting that the positive valence scenario (*p* < 0.001), whereas the opposite effect was observed when arousal was predicted to be high (*p* < 0.001). The interaction contrast indicated that this difference in differences was itself significant (*p* < 0.001).

Proportion of time spent lying was significantly affected by valence (*χ*^2^_1_ = 75.69, *p* < 0.001) and arousal (*χ*^2^_1_ = 78.04, *p* < 0.001, Fig. 11), but not the interaction between the two (*χ*^2^_1_ = 6.011, *p* = 0.014). Dogs spent significantly less time lying during both high arousal scenarios than the corresponding low arousal scenarios (*p* < 0.001). Dogs also spent significantly less time lying in the high arousal scenario designed to induce positive versus negative valence (*p* < 0.001). Although this effect was non-significant for the respective low arousal scenarios (*p* = 0.034), the interaction contrast indicated that the difference in these differences was itself non-significant (*p* = 0.054).

Proportion of time spent standing was significantly affected by valence (*χ*^2^_1_ = 62.34, *p* < 0.001), arousal (*χ*^2^_1_ = 170.8 *p* < 0.001), and the interaction between the two (*χ*^2^_1_ = 42.36, *p* < 0.001, Fig. 11). Dogs spent significantly more time standing in the high arousal scenario both when valence was anticipated to be positive (*p* < 0.001) and negative (*p* < 0.001). When high levels of arousal were induced, dogs spent significantly more time standing during the positive versus negative valance scenario (*p* < 0.001). However, when arousal was anticipated to be low, no significant difference in time spent standing was observed between scenarios predicted to induce positive or negative valence (*p* = 0.722). Further, the interaction contrast indicated that this difference in differences was itself significant (*p* < 0.001).

Proportion of time spent panting was significantly affected by both anticipated arousal (*χ*^2^_1_ = 35.50, *p* < 0.001), and the interaction between arousal and valance (*χ*^2^_1_ = 8.00, *p* = 0.005, Fig. 11), but not valence alone (*χ*^2^_1_ = 0.28, *p* = 0.599). Dogs spent more time panting during the high arousal scenario than the low arousal scenario when valence was anticipated to be negative (*p* < 0.001). However, no significant difference was observed between arousal levels when valence was anticipated to be positive (*p* = 0.070). No significant differences were observed between scenarios anticipated to induce positive or negative valence, whether anticipated arousal was high (*p* = 0.264), or low (*p* = 0.079). The interaction contrast indicated that the difference in these differences was itself significant (*p* = 0.019).

**Figure 12:**
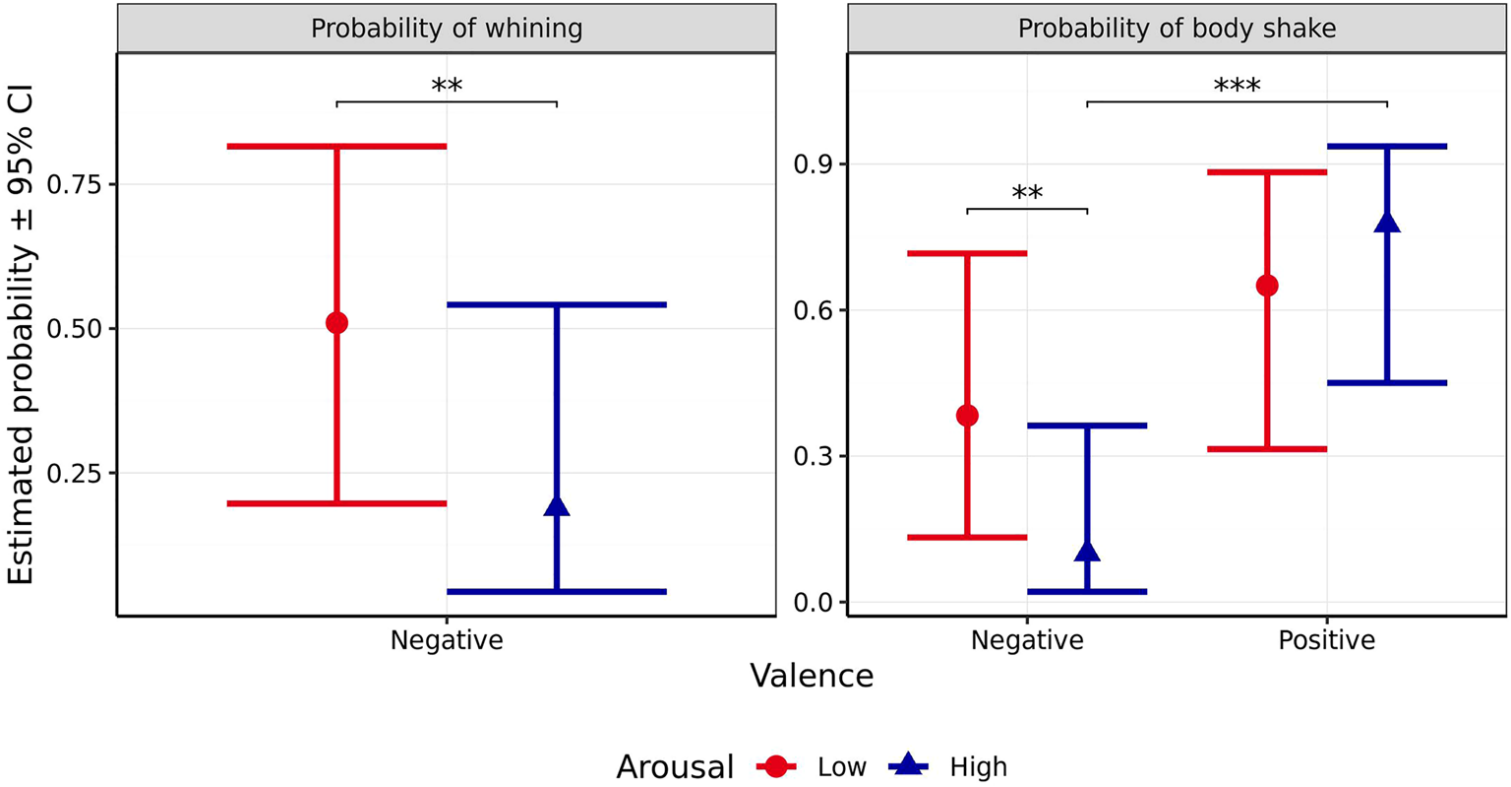
Estimated probability (± 95% CI) of performing body shake or whining behaviours during test scenarios anticipated to produce responses varying in valence (negative vs positive) and arousal (high vs low). These scenarios included those anticipated to produce negative valence low arousal (separation), negative valence high arousal (car), positive valence low arousal (petting), and positive valence high arousal (toy play). ** indicates a significant difference of *p* < 0.010. *** indicates a significant difference of *p* < 0.001.

Body shakes were observed infrequently and was only present within 43.8% of test scenarios. Its occurrence was therefore recorded as present/absent and it was subsequently treated as a binomial factor. Body shake behaviour was significantly affected by anticipated valence (*χ*^2^_1_ = 25.37, *p* < 0.001) and the interaction between valence and arousal (*χ*^2^_1_ = 11.12, *p* < 0.001, Fig. 12), but not arousal (*χ*^2^_1_ = 0.650, *p* = 0.420). When arousal was predicted to be high, body shakes were significantly more likely to occur in the positive versus negative valence scenario (*p* < 0.001). However, no significant difference between valence scenarios was observed when arousal was anticipated to be low (*p* = 0.044). When valence was anticipated to be negative, body shakes were significantly more likely to occur within the low arousal scenario compared to high (*p* = 0.007). However, no significant effect of arousal was observed when valence was anticipated to be positive (*p* = 0.409). The interaction contrast indicated that the difference in these differences was itself significant (*p* = 0.004).

Whining only occurred during four positive valence scenario test sessions. Therefore, it was only analyzed for negative valence scenarios. Furthermore, whining was only observed to occur within 33.9% of these negative valence test sessions and was therefore recorded as present/absent and treated as a binomial factor. For these scenarios, it was observed that whining was significantly more likely to occur when arousal was anticipated to be low compared to when it was anticipated to be high (*χ*^2^_1_ = 6.84, p = 0.009, Fig. 12), with the sole pairwise contrast providing the same *p*-value.

### Impact of food provision

As food was only provided during scenarios where valence was predicted to be positive, the impact of food on the parameters explored in this study could only be investigated with regards to the anticipated arousal of a scenario. Since the effect of arousal was analysed and reported previously, only effects relating to food provision and the interaction of food provision and arousal are reported here. Due to heteroscedasticity present in the residuals, a log-transformation was applied to the model for cortisol, serotonin, ACTH, sIgA, HRV (RMSSD and SDRR), and panting. All remaining outcome measures met model assumptions and proceeded without transformation. Estimated means and probabilities for different levels of arousal and food provision generated from these models are presented in Supplementary Table S2.

**Figure 13:**
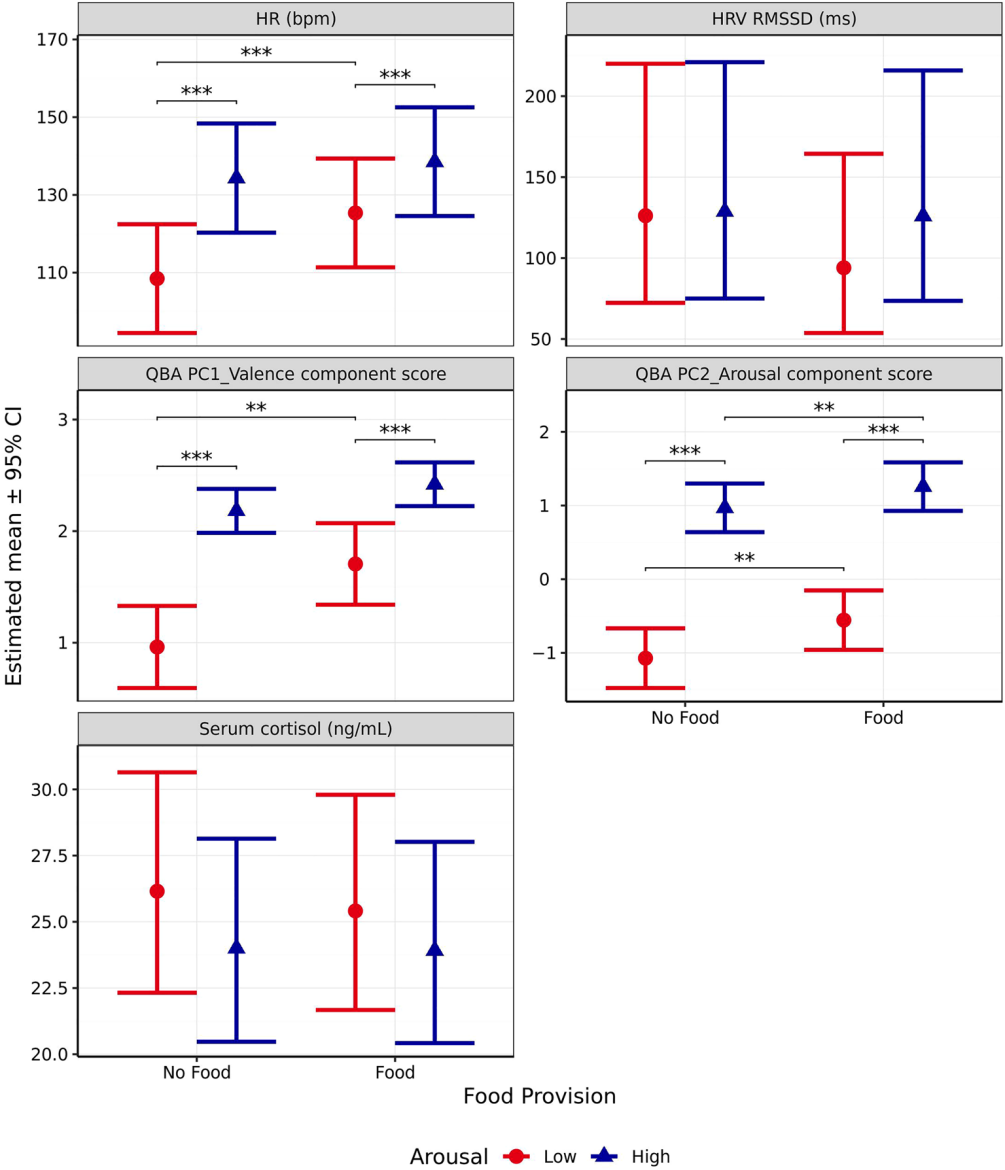
Estimated means (± 95% CI) for heart rate (bpm), heart rate variability measured using RMSSD (ms), PC1_Valence, PC2_Arousal, and blood serum cortisol (ng/mL) following test scenarios anticipated to produce positive valence responses varying in arousal (high vs low) and food provision (no food vs food provided). These scenarios included those anticipated to produce positive valence low arousal without food (calm petting), positive valence high arousal without food (toy play), positive valence low arousal with food (chew), and positive valence high arousal with food (treat-throwing). ** indicates a significant difference of *p* < 0.010. *** indicates a significant difference of *p* < 0.001.

Of the primary measures, no significant effect of food provision or the interaction between anticipated arousal and food was observed for serum cortisol or HRV-RMSSD (*p* > 0.01, Fig. 13). HR was significantly impacted by the provision of food (*χ*^2^_1_ = 49.24, *p* < 0.001) and the interaction between food and anticipated arousal (*χ*^2^_1_ = 17.50, *p* < 0.001, Fig. 13). HR was again found to be higher in high arousal scenarios than low arousal scenarios regardless of food provision (Food: *p* < 0.001; No food: *p* < 0.001). When arousal was anticipated to be high there was no significant difference in HR between scenarios including food and those that did not (*p* = 0.182), however, when arousal was anticipated to be low providing food resulted in a significant increase in HR (*p* < 0.001). Furthermore, the interaction contrast indicated that the difference in these differences was itself significant (*p* < 0.001).

Both PC1_Valence and PC2_Arousal were observed to be significantly higher for scenarios in which food was provided (PC1_Valence: *χ*^2^_1_ = 15.55, *p* < 0.001; PC2_Arousal: *χ*^2^_1_ = 17.57, *p* < 0.001), however, no significant interaction effect between food provision and anticipated arousal was observed (PC1_Valence: *χ*^2^_1_ = 5.369, *p* = 0.021; PC2_Arousal: *χ*^2^_1_ = 1.43, *p* = 0.232, Fig. 13). Both PC1_Valence and PC2_Arousal were found to be significantly higher in each pair of high versus low arousal conditions, irrespective of food provision (*p* < 0.001). PC1_Valence was found to be significantly higher in the low arousal condition in which food was provided, versus where it was not (*p* = 0.001), while the corresponding effect was found to be non-significant for high arousal scenarios (*p* = 0.034), although the interaction indicates the difference in these differences was also non-significant (*p* = 0.074). PC2_Arousal was found to be significantly higher when food was provided, for both low arousal (*p* = 0.008), and high arousal (*p* = 0.009) scenarios, with no significant interaction (*p* = 0.576).

**Figure 14:**
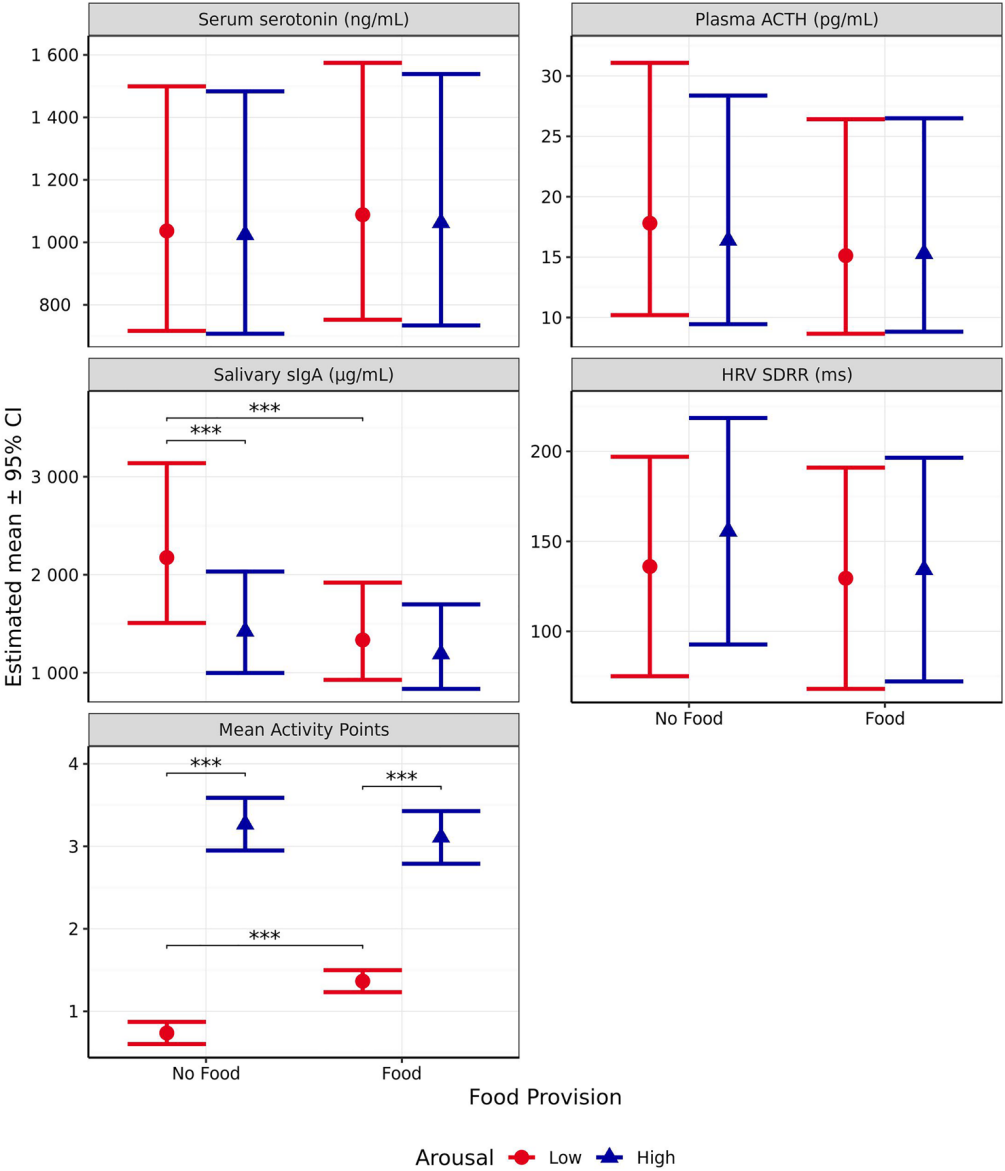
Estimated means (± 95% CI) for blood serum serotonin (ng/mL), plasma ACTH (pg/mL), salivary sIgA (µg/mL), heart rate variability measured using SDRR (ms), and mean Activity Points following test scenarios anticipated to produce positive valence responses varying in arousal (high vs low) and food provision (no food vs food provided). These scenarios included those anticipated to produce positive valence low arousal without food (calm petting), positive valence high arousal without food (toy play), positive valence low arousal with food (chew), and positive valence high arousal with food (treat-throwing). *** indicates a significant difference of *p* < 0.001.

No significant effect of food provision or the interaction between anticipated arousal and food was observed for serum serotonin, plasma ACTH, or HRV-SDRR (all *p* > 0.01, Fig. 14). However, the provision of food was found to significantly impact salivary sIgA (*χ*^2^_1_ = 27.80, *p* < 0.001). Furthermore, there was a significant interaction effect between anticipated arousal and the provision of food (*χ*^2^_1_ = 7.046, *p* = 0.008, Fig. 14). When food was provided, no significant difference in sIgA could be detected between the scenarios predicted to induce high and low levels of arousal (*p* = 0.434), whereas when food was not provided, low arousal resulted in an increase in sIgA compared to high arousal (*p* < 0.001). When arousal was low, providing food significantly reduced recorded sIgA (*p* < 0.001), although the same effect was non-significant when arousal was anticipated to be high (*p* = 0.059). The interaction contrast was not found to be significant (*p* = 0.031).

Mean Activity Points were significantly impacted by both the provision of food (*χ*^2^_1_ = 81.92 *p* < 0.001) and the interaction between food provision and anticipated arousal (*χ*^2^_1_ = 17.1, *p* < 0.001, Fig. 14). Activity Points were significantly higher for the high arousal scenarios than the low arousal scenarios regardless of whether food was provided (Food:* p* < 0.001; No Food: *p* < 0.001). When arousal was anticipated to be low, activity points were significantly higher when food was provided than when it was not (*p* < 0.001), whereas no significant effect of food provision was observed when arousal was anticipated to be high (*p* = 0.761). The interaction contrast indicated that the difference in these differences was itself significant (*p* < 0.001).

**Figure 15:**
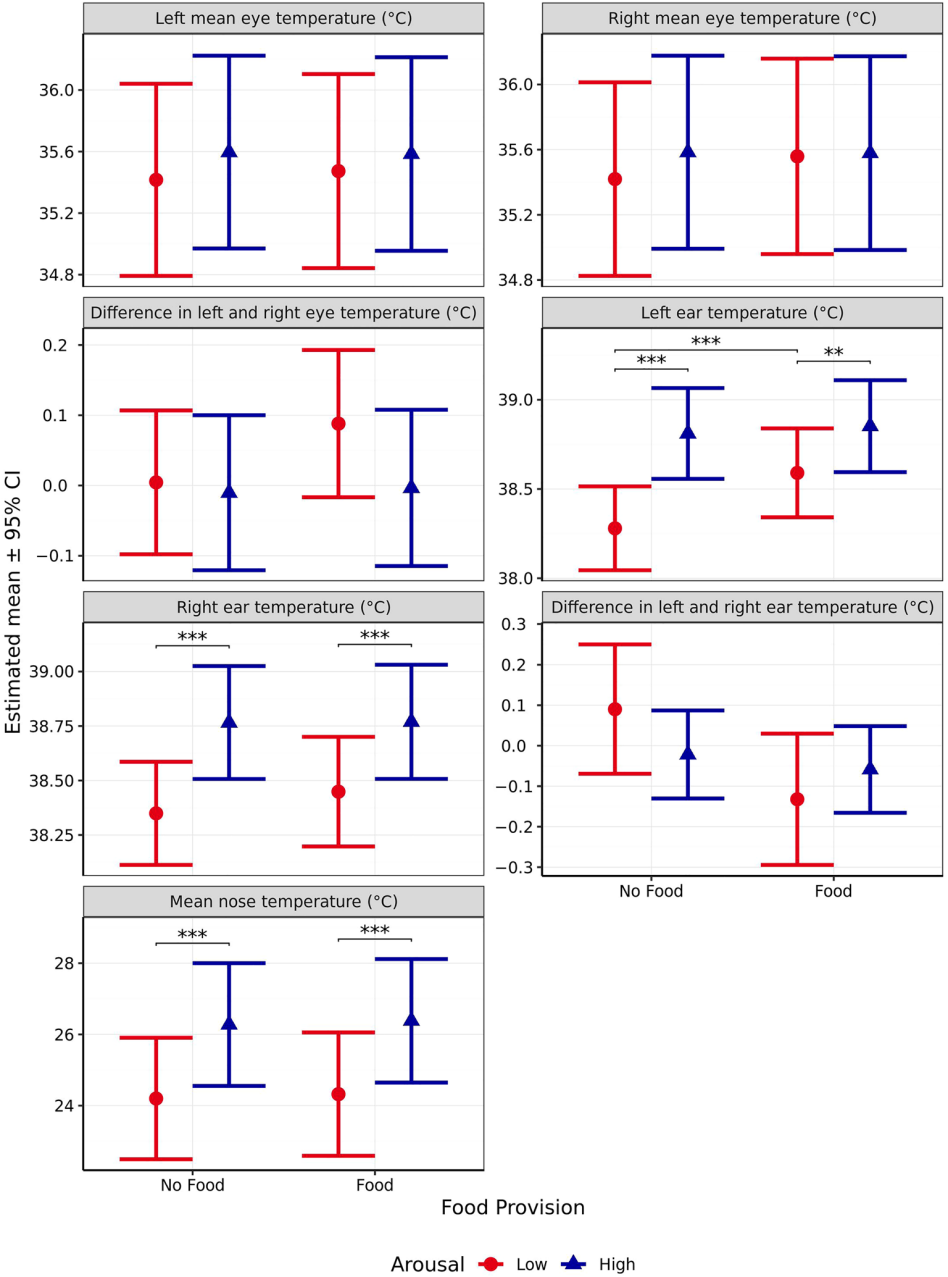
Estimated means (± 95% CI) for mean left eye temperature, mean right eye temperature, the difference in left and right mean eye temperature, left ear temperature, right ear temperature, the difference in left and right ear temperature, and mean nose temperature (all °C) following test scenarios anticipated to produce positive valence responses varying in arousal (high vs low) and food provision (no food vs food provided). These scenarios included those anticipated to produce positive valence low arousal without food (calm petting), positive valence high arousal without food (toy play), positive valence low arousal with food (chew), and positive valence high arousal with food (treat-throwing). ** indicates a significant difference of *p* < 0.010. *** indicates a significant difference of *p* < 0.001.

No significant effect of food provision or the interaction between anticipated arousal and food was observed for mean eye temperature (Left/Right/Difference), mean nose temperature, difference in ear temperature, or right ear temperature (all *p* > 0.01, Fig. 15). However, left ear temperature was significantly impacted by the provision of food (*χ*^2^_1_ = 14.43, *p* < 0.001) and the interaction between food and arousal (*χ*^2^_1_ = 6.584, *p* = 0.01, Fig. 15). It was again observed that the high arousal (positive valence) scenarios resulted in a higher left ear temperature than the low arousal (positive valence) scenarios, although this effect was more pronounced when food was not provided (Food: *p* = 0.002, No food: *p* < 0.001). When arousal was predicted to be high, the provision of food did not significantly impact left ear temperature (*p* = 0.947), whereas when arousal was predicted to be low, providing food resulted in a significantly higher left ear temperature (*p* < 0.001). The interaction contrast indicated that the difference in these differences was itself non-significant (*p* = 0.040).

**Figure 16:**
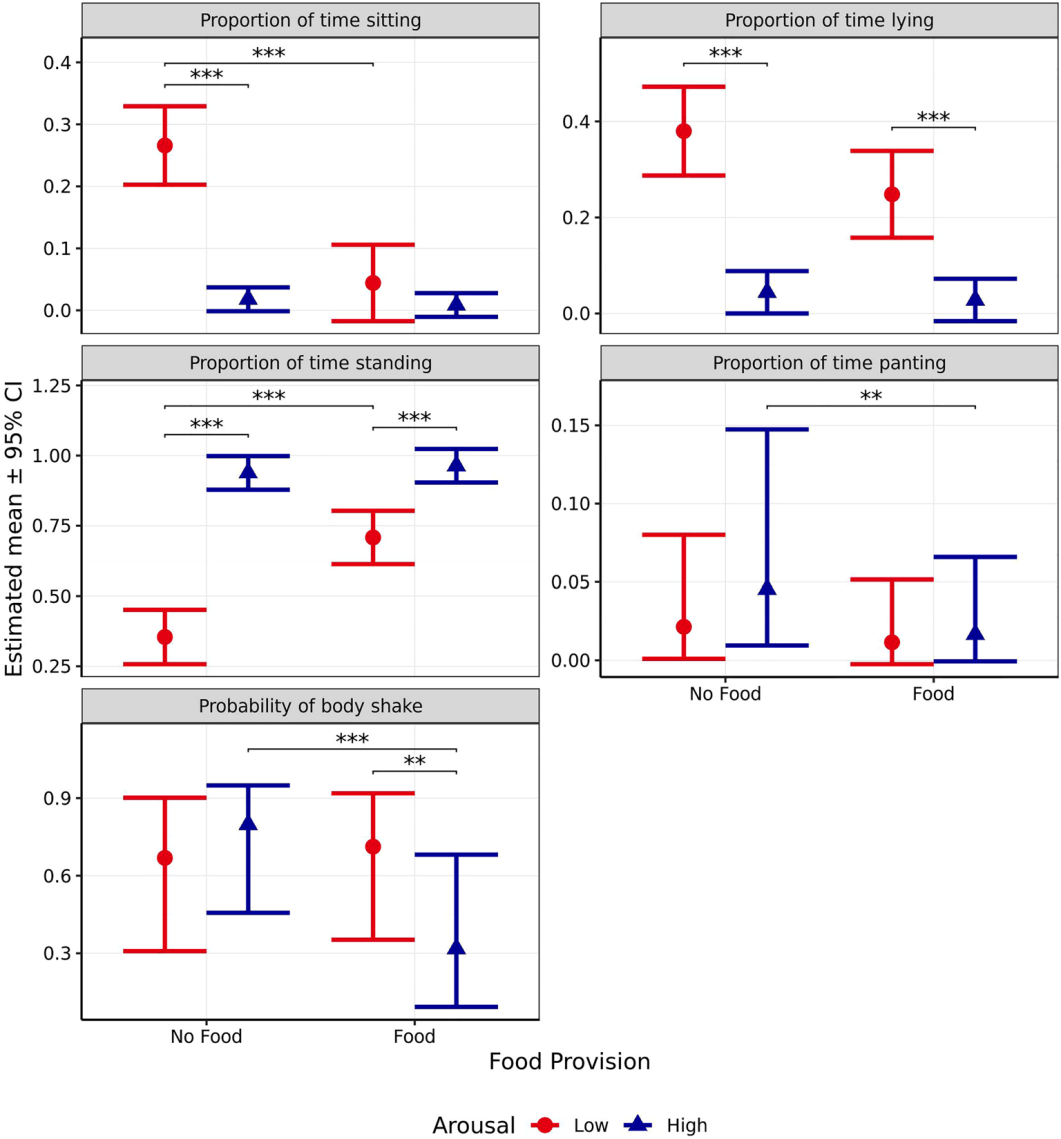
Estimated means (± 95% CI) of proportion of time spent sitting, proportion of time lying, proportion of time spent standing, proportion of time spent panting and probability of body shakes following test scenarios anticipated to produce positive valence responses varying in arousal (high vs low) and food provision (no food vs food provided). These scenarios included those anticipated to produce positive valence low arousal without food (calm petting), positive valence high arousal without food (toy play), positive valence low arousal with food (chew), and positive valence high arousal with food (treat-throwing). ** indicates a significant difference of *p* < 0.010. *** indicates a significant difference of *p* < 0.001.

No significant effect of food provision or the interaction between anticipated arousal and food was observed for proportion of time spent lying (all *p* > 0.01, Fig. 16). The proportion of the test session spent sitting was significantly influenced by both the provision of food (*χ*^2^_1_ = 6.984, *p* = 0.008) and the interaction effect between food and anticipated arousal (*χ*^2^_1_ = 36.59, *p* < 0.001, Fig. 16). When arousal was high no significant effect of food on time spent sitting was observed (*p* = 0.311). However, comparing low arousal scenarios, the provision of food resulted in dogs spending significantly less time spent sitting than when food was not provided (p < 0.001). Similarly, when food was provided, there was no significant difference in the duration of time spent sitting between the high and low arousal treatments (*p* = 0.407). However, when food was not provided, dogs spent a higher proportion of time sitting during the low arousal scenario than the high arousal scenario (*p* < 0.001). The interaction contrast indicated that the difference in these differences was itself significant (*p* < 0.001).

The proportion of the test session spent standing was significantly affected by the provision of food (*χ*^2^_1_ = 15.86, *p* < 0.001, Fig. 16) and the interaction between food and anticipated arousal (*χ*^2^_1_ = 47.69, *p* < 0.001). The proportion of time spent standing during a test scenario was significantly higher for high arousal scenarios than low arousal scenarios regardless of if food was provided (Food: p < 0.001; No Food: *p* < 0.001). When arousal was high, there was no significant effect of food on the proportion of time spent standing (*p* = 0.143), however when arousal was anticipated to be low, dogs spent significantly less time standing when no food was provided (*p* < 0.001). The interaction contrast indicated that the difference in these differences was itself significant (*p* < 0.001).

The proportion of the test session spent panting was significantly impacted by the provision of food (*χ*^2^_1_ = 14.37, *p* < 0.001, Fig. 16), with the provision of food resulting in significantly less panting. Pairwise contrasts indicated that this effect was significant between high arousal scenarios, for which significantly less panting was observed when food was provided (*p* = 0.002). While the same effect was non-significant comparing low arousal scenarios (*p* = 0.251), the interaction contrast indicated that the difference in these differences was non-significant (*p* = 0.581).

Once again, the occurrence of body shake behaviour during testing sessions was infrequent and therefore the parameter was transformed into a binomial factor for analysis. The probability of body shake behaviour occurring was significantly affected by the interaction between food and anticipated arousal (*χ*^2^_1_ = 12.87, *p* < 0.001, Fig. 16). When arousal was anticipated to be high, the provision of food was observed to significantly reduce the occurrence of body shake behaviour (*p* < 0.001). However, the provision of food had no significant impact on body shake behaviour when arousal was anticipated to be low (*p* = 0.954). Similarly, when food was provided, body shakes were significantly more likely to occur when the scenario was predicted to induce low versus high arousal (*p* = 0.001). No significant difference in likelihood of body shake behaviour was observed between scenarios predicted to induce high and low arousal when no food was provided (*p* = 0.367). The interaction contrast indicated that the difference in these differences was itself significant (*p* = 0.001).

## Discussion

The scenarios used in the current study were selected in a manner related to the a priori approach, as suggested by Mendl and colleagues^19^, but adapted to leverage both pre-existing and bespoke pilot data to empirically evaluate best fitting scenarios by quadrant in a more data-driven methodology. Comparison of ratings of valence and arousal levels provided by trained behaviour coders indicate that the actual responses of the dogs to the test scenarios were generally as anticipated, with high arousal, positive valence ratings being assigned to the treat throwing and toy play scenarios, and low arousal, positive valence ratings being assigned to the petting and long-lasting chew scenarios. Dogs were rated as responding with negative emotional valence, as anticipated, to both the car travel and separation scenarios, although the arousal ratings for both of these scenarios were similar. A possible explanation for this result is that raters may be more biased towards rating high arousal levels when more active behaviours are observed. This may be due to activity levels commonly being used as an indicator of arousal levels in dogs, e.g.^62,63^ despite high levels of emotional arousal also being possible when individuals are stationary. Due to the confined space of the car, dogs were not able to exhibit behaviours such as running and pacing, which are typically associated with high arousal and negative valence in dogs^64,65^. However, it is also possible individual dogs did not respond as anticipated due to individual differences in how the stimuli were perceived. This was likely influenced by experience, temperament and underlying mood state, therefore altering the resulting emotional response^19^. For example, frustration may have been induced in some dogs during the chew scenario if the dog finished the chew before the 10-min were completed and had to remain in the room while the handler was ignoring the dog. Additionally, dogs were not pre-screened for their response to separation, and may have responded with more high arousal anxiety than originally anticipated. This is supported by the fact that better separation based on arousal was observed during the pilot study than was observed in the main study. The dogs used in the current study were all raised and housed within a research facility, and had varying experience with the scenarios used based on their individual training histories, and participation in different research studies. It should also be noted that dogs that had a history of excessive destructive behaviour or resource guarding, and dogs who failed to habituate to sample collection or test areas, were excluded from the study for the purposes of both dog and human wellbeing. This could have potentially led to a biased population of dogs being tested and may have influenced some results. As no validated indicators of arousal and valence exist in dogs, and to avoid potential bias in arousal ratings influencing the results, the a priori categorization of valence and arousal levels were used for analysis. However, caution should be taken in interpreting the results related to arousal levels within the negative emotion scenarios.

The pattern of emotional response ratings observed during the current study follow a weak V shape, with higher arousal ratings being scored for more extreme ratings of valence. This is consistent with analyses from Kuppens et al*.*^66^, who concluded that the relationship between valence and arousal followed a weak asymmetric V-shape, with arousal increasing in response to both increasing positive and negative valence. It is important to consider this pattern in the interpretation of results relating to emotional valence and arousal, as it suggests the two dimensions may not be completely independent. Consequently, it may not be possible to reliably discriminate between parameters that are indicative of different levels of emotional arousal and those that are indicative of different magnitudes of valence within positive or negative emotional states.

### Indicators of arousal within negative emotional states

Cortisol, ACTH, HRV-RMSSD, panting, whining, and body shake all demonstrated significant differences based on anticipated arousal levels, but only within negative valence scenarios. Since there was a weak V shaped relationship between valence and arousal, it is possible these parameters are indicators of the magnitude of negative valence emotions as well as arousal. Therefore, these parameters are likely beneficial for use in studies aiming to identify the presence and/or intensity of negative emotional states in dogs. However, they are likely not beneficial for evaluating positive emotional states in dogs.

Cortisol and ACTH were both significantly higher for the high arousal negative valence scenario (Q2) compared to scenarios from any other quadrant. Further, cortisol was significantly higher for the low arousal negative valence scenario (Q3) in comparison to the positive valence scenarios. Cortisol and ACTH are both hormones involved in the HPA axis and play an integral part in the body’s response to stress. The HPA axis is first activated in the hypothalamus, resulting in release of corticotropin releasing hormone. This hormone acts on the anterior pituitary to stimulate the release of ACTH, which in turn triggers the release of cortisol from the adrenal cortex^67–69^. Cortisol, or its metabolites, are widely used in animal welfare research, and can be successfully measured in the blood plasma, saliva, urine, feces, or hair of dogs, e.g.^70–75^. Numerous studies have demonstrated that cortisol increases in situations presumed to cause stress, fear, and anxiety in dogs, suggesting it to be a useful indicator of negative emotional state, e.g.^10,11,37,70,76^. This increase has also been demonstrated within dogs during positive interactions with their owners^77^, indicating cortisol may also fluctuate with arousal in positive emotional states. A study looking at different populations of working dogs found cortisol decreased following play sessions in one population, but increased in the other population^78^. It should be noted that these populations likely differed in how positively the dogs interpreted the play as the authors noted a qualitative difference in the type of play the dogs were engaged with. Cortisol was observed to decrease in the population experiencing more spontaneous play, while cortisol increased in the population that received commands during play^78^. Results from the current study suggest that cortisol and ACTH are both beneficial indicators of negative emotional states and can be used to quantify different emotional intensity within negative emotions, but do not significantly vary with arousal within positive states. Within the current study, all pairwise differences were more pronounced for measurements of cortisol compared to ACTH. This may be due to ACTH initially surging in response to stress, followed by a slower rise in cortisol^79^, resulting in noise being introduced to ACTH measurements caused by stress related to handling during post-test sampling. Additionally, ACTH release is inhibited by cortisol^67^, which likely contributed to reduced levels of the hormone being measured by the time of sampling. Consideration of this modulation of ACTH and cortisol over time should be taken when selecting which is the most appropriate parameter for a given study design based on the duration of the emotion-eliciting event, and latency until the sample is taken. Furthermore, these results suggest that neither cortisol nor ACTH should be considered for quantifying positive emotional states.

Another parameter that differed significantly within negative valence scenarios, but not positive valence scenarios was HRV-RMSSD, with higher values observed during the low arousal negative valence scenario, compared to all other scenarios. Further, when measured as SDRR, HRV was significantly higher in the negative valence low arousal scenario when compared to the positive valence low arousal scenario. HRV is a measure of the variance in time intervals between heartbeats and is reflective of the balance between the sympathetic and parasympathetic branches of the autonomic nervous system^80–82^. The sympathetic system is responsible for fight or flight response, and increases in physical and affective readiness, while the parasympathetic system is responsible for decreased excitation during periods of rest^83^. As a measure of the balance between these two systems HRV has been suggested to be an indicator of emotional valence and arousal^84^. Additionally, HRV may be less influenced by body position and physical activity than other heart rate based parameters^85^. However, HR and HRV have been shown to be correlated, with lower HRV being observed when HR is high^86,87^. There are multiple different methods of measuring and calculating HRV, which may be more or less appropriate depending on the objective of the study^86^. Results from previous research using HRV parameters to measure stress in dogs have varied in their results depending on the situation and specific HRV parameter used, e.g.^84,88,89^. A majority of the research in dogs using RMSSD to measure HRV has identified a decrease in response to negative situations^84,90,91^. One study in particular demonstrated that RMSSD decreased in negative valence scenarios in dogs, but did not change in response to positive valence^84^, which is in contrast to results of the current study where HRV-RMSSD was higher in negative valence scenarios. Conversely, SDNN, a similar time-domain based HRV parameter to SDRR, with removal of non-normal beats, decreased in response to the positive valence scenarios, but did not change in response to the negative valence scenarios^84^. These results are similar to the results of the current study, where HRV-SDRR was lower in positive valence scenarios compared to negative valence scenarios. The scenarios used to elicit positive and negative valence in that study were similar to those used in the current study, with the positive valence scenario consisting of the owner petting their dog, and the negative valence scenario consisting of a period of separation.. One explanation for this difference could be the dog’s attentive state with dogs focusing on the door after being left by their handler. It has been suggested that periods of increased attention are positively associated with HRV^85,92^. For example, HRV measured using SDNN has been shown to increase when dogs are focused on a door after the departure of their owner^85,92^ or when focused on a ball^92^. However, it is unknown why the same effect was not observed in the positive valence scenarios used during the current study (i.e., toy play and treat throwing), which could also be expected to induce higher attentive states. It is possible that these conflicting results may be the result of measurement error during this study, as there were some dogs that experienced ECG data loss due to connectivity issues with the ECG nodes. Artefacts in the ECG readings introduced by movement and activity may have also impacted on the calculated HRV^86^, especially when measuring HRV using R-R intervals without removal of non-normal beats. This potentially contributed to significant findings in HRV during the low arousal scenarios and not the high arousal scenarios. Additionally, other factors such as breed, individual variation, physical activity and respiration have been shown to influence HRV^86,93,94^, and may have contributed to these results. These results indicate that HRV may be a useful indicator of emotional valence, but primarily within low arousal scenarios. Caution should be taken to ensure the quality of readings, especially in situations where the dog is in motion.

The occurrence of certain behaviour parameters, namely panting, whining, and body shakes, were all significantly associated with anticipated arousal, but only within negative valence scenarios. Panting was significantly higher in the high arousal negative valence scenario compared to the low arousal negative valence scenario. Panting serves as a method of thermoregulation in dogs, allowing for evaporative cooling in response to heat stress or physical exertion^95^. However, this behaviour has also been demonstrated to increase with emotional stress in dogs, e.g.^96–101^. As there were no significant differences in activity between the high and low arousal negative valence scenarios, and the car and test room were maintained at a constant temperature throughout the study, it can be concluded that the differences in panting observed in the current study are likely due to emotional stress. Notably, in the current study, panting was not significantly higher in the high arousal positive valence scenarios, which involved engaging the dog in play with a toy, despite dogs showing significantly higher activity levels (as measured with the activity monitor) during this scenario. However, it is possible panting was inhibited in this scenario due to the presence of a toy in the dog’s mouth. Panting was also significantly lower for the high arousal positive valence scenario involving food compared to the high arousal positive valence scenario without food. Similarly, dogs may have prioritized other mutually exclusive behaviours, such as mouth-closed anticipation, seeking, and consumption behaviours over panting in food-based scenarios. It is therefore possible that panting is not a specific indicator of negative emotional state and should be interpreted in the context in which it occurs and in combination with other parameters, such as activity and temperature.

Whining was only able to be analysed in the negative valence scenarios, due to rare occurrence in the positive valence scenarios. Within negative valence scenarios, whining was significantly higher in the low arousal scenario (i.e., separation) compared to the high arousal scenario (i.e., car travel). It is possible that the difference in whining between these two scenarios may have been due to the specific scenarios being used. Whining is often considered a social signal of distress and has been shown to increase in response to separation from an attachment figure^63,102–105^, as well as during periods of frustration when desired resources are withheld^106,107^. Furthermore, whining is often interpreted as being indicative of negative valence by both dog owners and non-owners, with similar ratings of sadness being attributed to dog whines as to human infant distress cries^108^. Based on these results, whining appears to be a useful behavioural indicator of negative emotional states, although it is most likely restricted by the social context in which it is performed. Further research is required to understand how whining varies across negative emotional states in different contexts.

The probability of dogs performing a body shake was significantly lower in the high arousal negative valence scenario compared to any other quadrant. Body shakes are hypothesized to be displacement behaviours linked to anxiety and stress^10,11,109^, and have been argued to relieve tension accumulated over a period of time^97^. In the current study, dogs may have performed this behaviour to relieve tension built up during high arousal toy play. However, other factors may have also contributed to observation of body shakes. For example, dogs may have performed this behaviour to resettle their fur after stimulation during petting. Further, dogs may have performed this behaviour more frequently in the current study due to minor irritation or stimulation caused by wearing the multi-parameter harness. During the high arousal negative valence scenario, dogs were confined to a crate in an unstable environment (i.e., moving car) which may have inhibited their ability to shake-off. Interestingly, lower probabilities of body shakes were also observed in the high arousal positive valence scenario when food was provided (i.e., treat throwing), compared to the low arousal positive valence scenario with food (i.e., long-lasting chew) and the high arousal positive valence scenario without food (i.e., toy play). This may be due to the dogs' focus on the treats, causing them to freeze in anticipation of the next piece of food to be thrown. However, the context in which the body shakes were performed was not coded, and therefore the immediate antecedent for these behaviours cannot be determined. Previous research has found inconsistent results when measuring body shakes as an indicator of stress. While some studies observed an increase in response to stressful events^10,11,97,109,110^, others found the behaviour to be infrequent, or identified no significant changes in frequency due to stress^100,111–114^. It is worth considering that one study looking at potential guide dog puppies found poor test re-test reliability for using body shakes over time^115^. However, that study did find that dogs performing a body shake following a standardized handling test at one time point was predictive of successful qualification as a guide dog^115^. The authors suggested this may indicate that body shakes are part of an effective coping response to minor stressors^115^. Therefore, this behaviour may not be a specific indicator of emotional state and should be used with caution and in combination with other parameters. Additionally, future research should consider the circumstances surrounding the performance of body shakes, such as the immediate antecedents to the behaviour.

### Indicators of arousal within positive emotional states

The parameter that appeared to provide the clearest indication of positive emotional states in the current study was QBA PC1_Valence scores. These component scores were higher in the scenarios that were anticipated to induce positive valence in comparison to those anticipated to induce negative valence. Additionally, within scenarios that were anticipated to generate positive valence, PC1_Valence scores were significantly higher when arousal was high, in comparison to when arousal was low. This pattern of results following an asymmetric V-shape, with higher valence ratings for higher levels of arousal, especially within positive valence, is consistent with those identified using the valence and arousal ratings within this study, as well as analyses reported by Kuppens et al*.*^66^. Similar results have been found in other studies looking at a range of species, where high arousal is associated with more extreme valence scores and is hypothesized to be related to the more active behaviours, e.g.^31,116^. However, when valence was anticipated to be negative, there was no significant difference in PC1_Valence scores between the high and low arousal scenarios. These component scores were based on subjective ratings provided by trained coders, and may therefore have been subject to observer biases^117^. While the analyzed scores met acceptable levels of both inter- and intra-rater reliability, it is possible that they were not reflective of the dogs’ true internal state. Additionally, the terms ‘alert’, ‘bored’, ‘explorative’, ‘fearful’, and ‘frustrated’ were excluded from analysis due to issues with reliability. As a number of these terms are reflective of negative valence emotions, covering low (bored) and high (fearful, frustrated) arousal this may have impacted the ability of the QBA to differentiate arousal in negative valence scenarios. Similarly, the terms ‘bored’, ‘alert’, ‘explorative’, and ‘fearful’ have also been found to have poor inter-rater reliability in a study on shelter dogs^22^. Another study also on shelter dogs found that the term ‘bored’ was not rated reliably between raters^118^. Additionally, ‘bored’ and ‘frustrated’ have received poor inter-rater reliability scores in studies on multiple species, e.g.^24,119^. These studies, as well as our findings, suggest that the terms discussed may be difficult to interpret, even for trained raters. Boredom and frustration may be particularly challenging to interpret as they are reliant on context as well as behaviour. Due to the types of scenarios used in this study, it was not possible to blind the coders to the scenario during coding. It is therefore possible raters assigned higher valence ratings for the scenarios they anticipated to be more rewarding to the dog (i.e., toy play, provision of chew/treats, petting). Despite this, numerous studies across a range of species and situations have demonstrated associations between QBA scores and physiological parameters, e.g.^38,39,120–122^. However, some studies have also failed to find an association^123,124^. Based on these results we recommend QBAs as a useful tool for quantifying emotional valence, however, use in combination with other objective behavioural and physiological parameters would be beneficial.

A number of additional parameters also demonstrated significant differences based on arousal, but only within positive valence scenarios. These included QBA PC2_Arousal scores, activity level, ear temperature, and the proportion of time spent sitting. While this lack of significant differences in arousal within the negative valence scenarios may indicate that they were not successful in inducing differences in emotion based on arousal levels, results of the physiological parameters suggest this is not the case. Cortisol, ACTH, and HR were all elevated for the high arousal negative valence scenario when compared to the low arousal negative valence scenario. It is therefore more likely that these parameters (i.e., QBA PC2_Arousal scores, activity level, ear temperature, proportion of time spent sitting) are only effective for quantifying arousal within positive emotional states. However, it is worth noting that these parameters may have been influenced by higher levels of activity elicited by the high arousal positive valence scenarios due to the nature of the scenarios used. A similar pattern was observed in the valence and arousal ratings, with differences based on arousal ratings present in the positive valence scenarios, but not the negative valence scenarios. As discussed previously, this may be due to coders being biased towards scoring arousal higher when dogs displayed more active behaviours. This bias may also have impacted the PC2_Arousal ratings, as the QBA scores used to calculate it were completed by the same coders as the valence and arousal scores. This is supported by the fact that activity levels were higher and time spent sitting was lower in the high arousal positive valence scenarios compared to any other scenario. This is likely due to a combination of activity increasing with arousal levels and activity being encouraged as part of the positive valence scenarios (i.e., dogs were engaged with play with toys). Further, movement was restricted within the higher arousal negative valence scenario as the dog was confined to a crate within a car. Based on these results, caution should be taken using the parameters of PC2_Arousal and activity as indicators of arousal, especially in scenarios where activity is specifically encouraged or inhibited.

Increases in activity likely also contributed to the higher ear and nose temperatures observed in the high arousal positive valence scenario. The performance of exercise leads to an increased metabolic heat production, resulting in higher body temperatures being observed^125–127^. Furthermore, multiple studies have highlighted either a relationship between activity level and body temperature or a strong similarity between the circadian rhythm of the two^128–131^. Additionally, caution should be taken when interpreting temperature data collected through the use of infra-red thermography, as experimental set-up and environmental factors, such as camera-object distance and external temperature, have been documented to impact the accuracy of infra-red thermography readings^132–135^.

In the current study surface temperatures of the nose and eye were significantly lower for the high arousal negative valence scenario compared to the high arousal positive valence scenario. This is contrary to previous findings, which have indicated that temperature increases in response to stress^39,96,113,136^. While testing and sampling areas were maintained at consistent temperatures throughout the study, dogs had to walk outside from the car to the sampling room prior to collection of the temperature readings. This period of exposure to outside temperature (which were lower than those experienced inside for a majority of the study period) likely contributed to the lower eye and nose temperatures observed following the car test. This is highlighted by the fact that outside temperature was a significant factor in a number of the models involving temperature parameters. Care should be taken to ensure animals are kept within temperature-controlled environments for a suitable period of time prior to collection of temperature measurements, especially if infra-red thermography is used. Furthermore, additional parameters such as environmental temperature and animal activity levels should be considered with any temperature analysis.

Finally, dogs spent less time sitting in the high arousal positive valence scenario, compared to the low arousal positive valence scenario. While this is likely influenced by the higher levels of activity observed in the high arousal scenario, it is also possible higher levels of sitting were observed in the low arousal scenario due to the nature of the scenario and the dogs previous training history. In this scenario dogs were being pet by a handler who was sitting on the floor. They may therefore have been more likely to sit in this scenario as a conditioned response to solicit attention, or as sitting provided the most comfortable position for the dog during petting. Sitting may, therefore, not be an effective indicator of emotional valence and may instead be context specific.

### Indicators of arousal

HR, sIgA, standing, and lying all showed consistent changes associated with arousal regardless of valence, with this effect being more pronounced within the positive valence scenarios. This is likely due to the difference in actual arousal levels induced being more pronounced between the positive valence scenarios than the negative valence scenarios. It may also have been the result of higher levels of activity occurring within the positive valence scenarios. These parameters have the potential to be useful indicators of emotional arousal, and when used in combination with indicators of emotional valence, can provide additional information on what an animal may be feeling.

Like HRV, HR is controlled by the autonomic nervous system, and has been shown to increase in response to stress in dogs, e.g.^10,12,38,109,137^. However, HR can also be affected by additional factors, such as physical activity, body posture, external environment, and eating, impacting its reliability as an indicator of emotional state^36,85,137,138^. In the current study, HR increased with high arousal in both positive and negative valence scenarios. This effect was more pronounced when valence was anticipated to be positive, although it should be noted that this scenario also induced higher levels of activity, which may have contributed to higher HR levels. Interestingly, HR also increased with higher arousal in the negative valence scenario, despite similar levels of activity being observed, suggesting emotional stress was the primary driver of this increase. When looking at the effect of food provision, HR was significantly higher for the low arousal scenario when food was provided compared to the low arousal scenario without food. Within high arousal scenarios, there was no effect of food provision. This may indicate that the process of food consumption caused a spike in HR, as reported by Kostarczyh and Fonberg^36^. However, this effect may have also been caused by these two events not being equally low in arousal, which appears to be reflected in the other study parameters. For example, higher mean Activity Points, PC1_Valence scores, PC2_Arousal scores, left ear temperature, and time spent standing, and lower time spent sitting and lying were observed for the low arousal scenario with food (i.e., long-lasting chew) compared to the low arousal scenario without food (i.e., petting). Further, higher PC2_Arousal scores were observed in the high arousal scenario with food (i.e., treat throwing) when compared to the high arousal scenario without food (i.e., toy play). Combined, these results suggest that the dogs found the scenarios with provision of food more arousing than scenarios without food. These findings should be considered in the selection of appropriate interventions for research purposes, and may also be of relevance in practical situations where food is provided for the purposes of training and/or behaviour modification. Consideration of how arousal levels may influence the dog’s ability to learn and perform desired behaviours^139^ should be made alongside the selection of appropriate rewards and reward delivery methods. However, as only two food scenarios were tested in the current study, caution should be taken in extrapolating these findings more broadly.

Another parameter that appears to be associated with arousal in the current study is sIgA. Secretory IgA plays a key role in the immune system and primarily functions within the mucous membranes protecting against infectious agents and pathogens^140^. Studies in dogs have demonstrated that sIgA decreases in response to both chronic^141^ and acute^142,143^ stress. However, increases in response to acute stress have also been observed^39^. This may be partially due factors such as breed, age, time of day, and individual variation, which have been demonstrated to significantly influence sIgA values^142,144,145^. Further, it is suggested that a number of additional factors, such as collection material, salivation rate, and food contamination may impact results of salivary parameters^146,147^. While sIgA was found to decrease in response to high arousal activities in the current study, this occurred in both negative and positive valence scenarios. Additionally, the provision of food also resulted in a decrease in sIgA levels in the low arousal, positive valence scenario. It is possible that the changes in sIgA concentrations observed in the current study are not indicative of emotional state, but are instead influenced by changes in salivation rate caused by panting, appetitive behaviour, and/or physical exertion. Further research is required to understand the relationship between sIgA and emotional states, as well as the impact of different external factors, such as salivation and the provision of food.

Finally, standing and lying were significantly associated with arousal in the current study, with higher levels of standing and lower levels of lying observed in the high arousal scenarios. This was as anticipated, as dogs were more active during the high arousal positive valence scenarios. Furthermore, this demonstrates that while the high arousal negative valence scenario did not result in higher activity levels, the dogs were less settled and willing to lie down during this scenario. However, it is unknown whether this was due to emotional stress or the need to physically brace against movements of the car as it performed the stopping and turning manoeuvres as part of the test route.

When combined with indicators of valence, these indicators of arousal would be beneficial in providing a more detailed view of emotional state, further quantifying the emotional intensity within positive and negative states. However, potential confounding factors, such as exercise, external temperature, and food provision should be considered when selecting the most appropriate parameter for the study design.

### Other parameters

The only parameters explored in this study that were not associated with valence and arousal were serotonin and the difference between left and right temperatures of the eye and ear. Serotonin, and the serotonergic system as a whole, are thought to play a role in anxiety modulation, with a range of evidence showing both anxiolytic and anxiogenic effects^148^, as well as functions in reward processing^149^. In dogs, serotonin has been negatively correlated with aggression^150–153^, anxiety^154,155^, and cortisol levels^150,154^. However, based on the current results it is unlikely serotonin levels change in response to acute changes in emotional state. As mentioned previously, dogs with a history of destructive behaviour or resource guarding, and those who were unable to habituate to sample collection or the testing environment, were excluded from the study. This may have biased the study population away from dogs with behavioural issues related to imbalances in serotonin. It is possible that significant effects of serotonin may be observed if a population of dogs with behavioural issues was targeted. Further exploration into the relationship between serotonin and more chronic emotional states is warranted.

The difference in temperature between the left and right ear and eye were included in the present study based on the hypothesis that hemispheric lateralization of emotional processing in the brain would result in an asymmetry in temperatures between the left and right eye and ear. Hemispheric lateralization occurs when one hemisphere of the brain has higher requirements of energy to process a task or stimulus, affecting the cerebral blood flow^156,157^. This altered blood flow has been shown to create a temperature difference between the tympanic membranes of human participants during cognitive tasks^156,158^. Temperature disparities between left and right tympanic membranes were also found in stress-induced cats, with higher right temperatures in cats with high cortisol levels and higher left tympanic temperatures with low cortisol levels^159^. These studies indicate a lateralization of emotion processing, suggesting this may be an appropriate indicator of emotional valence or arousal. However, this hypothesis was not supported by the results of the current study. This may indicate that emotions elicited in the current study were not sufficient in intensity and/or duration to induce measurable asymmetry in temperature. Alternatively, the current results may support the hypothesis that emotion processing is not consistently lateralized to either the left or right hemisphere, and is instead a dynamic process incorporating multiple interrelated networks, which may be left- or right-biased, or bilateral in activation^160^.

It is worth noting that any differences in temperature may have been too small to be detected due to the sensitivity of the measurement instruments. While the infra-red camera has excellent sensitivity for detecting differences within a frame (~ 0.03 °C), it is still possible minor variations in the distance between each eye and the camera, and the influence of hair obstructing a portion of the eye may have influenced the mean temperature reading. The ear thermometer was more variable with potential technical deviation of ± 0.2 °C, and accurate measurements are reliant on correct thermometer insertion techniques. Furthermore, obstruction or contamination of the tympanic membrane during insertion has been shown to result in inaccurate readings^161^.

## Conclusions

The results of this study highlight that no single parameter in isolation can be used to quantify both valence and arousal across all emotional quadrants. This further emphasizes the importance of a multi-parameter approach for evaluating animal emotions^162,163^. While scores from the QBA showed the most differentiation both within positive emotional states, and between positive and negative emotional states, there are some limitations to this approach, as less actively expressed emotions are likely not well captured. Furthermore, ratings may have been influenced by observer biases. Meanwhile, cortisol, ACTH, HRV-RMSSD, panting, whining, and body shake showed promise as potential indicators of negative emotional states, but did not differentiate within positive valence emotions. These parameters are therefore recommended for identifying the presence of negative emotional states, or quantifying arousal within negative emotional states. HRV (RMSSD and SDRR) was able to successfully differentiate between positive and negative valence, however only within low arousal scenarios. This parameter was likely influenced by noise introduced in scenarios with high levels of activity, and therefore caution should be taken to ensure the quality of readings used in future studies. Other measures, including HR, sIgA, and body position were found to be indicative of arousal levels, regardless of valence. It should be noted that several parameters, including HRV, sIgA, body shake, panting, ear and eye temperatures, body position and activity appeared to be affected by external factors, such as food provision, temperature, exercise, and confinement, and should be utilised with caution based on the study design to be used when assessing dog emotion. Finally, serotonin and laterality of ear and eye temperatures were not successful in the current study and are likely not useful indicators of acute emotional states in dogs. Overall, it is recommended that researchers use a combination of parameters including indicators of both valence and arousal which can be selected based on the emotional quadrants being targeted, and the limitations of the study design. These results provide a critical first step towards identifying evidence-based indicators of short-term emotional states in dogs and could be used to provide more holistic welfare assessments. However, it should be highlighted that parameters for the current study were selected based on hypotheses generated from previous literature, which has primarily focused on negative emotional states. It is likely additional parameters exist that are accurate and reliable indicators of positive emotional states that have yet to be fully understood and explored.

### Supplementary Information

Supplementary Tables.

## Data Availability

The datasets used and/or analyzed during the current study are available from the corresponding author upon reasonable request.
