# Supplementary material for: Evaluation of indicators of acute emotional states in dogs
Source: Sci Rep. 2024 Mar 17;14:6406. doi: 10.1038/s41598-024-56859-9 (PMC10944520; doi:10.1038/s41598-024-56859-9)
Supplement: Supplementary file 1 — Supplementary Tables. [file 41598_2024_56859_MOESM1_ESM.pdf]

Supplementary Information for

Evaluation of indicators of acute emotional states in dogs

Hannah E. Flint, Jennifer E. Weller, Nia Parry-Howells, Zack W. Ellerby, Stephanie L. McKay, Tammie King

This file contains:

Table S1 – Results of model pertaining to the effect of anticipated arousal and valence on parameters

Table S2 - Results of model pertaining to the effect of anticipated arousal and the provision of food on parameters

Table S1. – Estimated means/estimated probability ($\bar{x}$) and upper/lower 95% confidence intervals (CI) for contrasts explored within models pertaining to effect of anticipated arousal and valence on primary and secondary parameters. Matching superscript letters within a parameter indicate significant difference in means based on model contrasts (α=0.010).

				**Valence**					
				Positive			*Negative*		
**Parameter**	**Unit**	**n**	**Arousal**	$\bar{x}$	***Upper 95% CI***	***Lower 95% CI***	$\bar{x}$	***Upper 95% CI***	***Lower 95% CI***
Cortisol	*ng/ml*	204	*High*	24.051^{a}	28.160	20.542	59.735^{ac}	70.013	50.965
			Low	26.334^{b}	30.107	23.034	34.529^{bc}	39.409	30.252
Heart Rate	*bpm*	185	*High*	133.777^{ac}	144.657	122.898	121.151^{ad}	132.019	110.283
			Low	108.452^{c}	118.998	97.906	105.643^{d}	116.194	95.091
Heart Rate Variability (RMSSD)	ms	191	*High*	132.732	188.415	93.504	118.359^{b}	167.631	83.569
			Low	128.761^{a}	177.498	93.405	176.621^{ab}	243.891	127.905
PC1_Valence	-	231	*High*	2.187^{ac}	2.529	1.844	-3.573^{a}	-3.230	-3.915
			Low	0.963^{bc}	1.327	0.598	-3.496^{b}	-3.129	-3.863
PC2_Arousal	-	231	*High*	0.995^{ac}	1.265	0.725	0.116^{a}	0.386	-0.153
			Low	-1.037^{bc}	-0.679	-1.394	0.002^{b}	0.361	-0.358
Serotonin	ng/mL	201	*High*	1029.377	1503.726	704.660	1078.104	1576.072	737.471
			Low	1036.688	1513.019	710.315	1047.071	1527.485	717.753
ACTH	pg/mL	199	*High*	16.611^{a}	25.936	10.637	31.934^{ab}	49.947	20.416
			Low	17.979	27.536	11.738	19.007^{b}	29.102	12.412
sIgA	µg/mL	198	*High*	1381.987^{a}	990.2717	1928.65	1499.153	1073.122	2094.319
			Low	2069.414^{a}	1489.077	2875.924	1908.588	1374.296	2650.597
Heart Rate Variability (SDRR)	ms	191	*High*	156.107	201.258	110.956	136.017	179.549	90.485
			Low	136.041^{a}	171.227	100.856	163.733^{a}	199.114	128.352
Left Eye Mean Temp	°C	220	*High*	35.489^{a}	36.056	34.922	35.095^{a}	35.660	34.530
			Low	35.315	35.876	34.753	35.380	35.945	34.815
Right Eye Mean Temp	°C	220	*High*	35.414^{a}	35.967	34.861	35.068^{a}	35.619	34.517
			Low	35.263	35.806	34.720	35.299	35.845	34.753
Eye Mean Temp Difference	°C	220	*High*	-0.010	0.115	-0.134	0.032	0.162	-0.098
			Low	0.010	0.106	-0.087	-0.018	0.079	-0.116
Left Ear Temp	°C	224	*High*	38.795^{ab}	39.056	38.535	38.328^{a}	38.587	38.069
			Low	38.273^{b}	38.490	38.055	38.423	38.647	38.199
Right Ear Temp	°C	222	*High*	38.747^{ab}	39.004	38.489	38.273^{a}	38.530	38.016
			Low	38.330^{b}	38.534	38.126	38.371	38.581	38.161
Ear Temp Difference	°C	222	*High*	-0.014	0.124	-0.152	-0.008	0.134	-0.150
			Low	0.089	0.237	-0.059	-0.018	0.131	-0.167
Nose Mean Temp	°C	220	*High*	25.544^{ab}	27.089	23.998	22.309^{ac}	23.846	20.771
			Low	23.552^{b}	25.014	22.089	23.448^{c}	24.926	21.969

Mean Activity Points	-	217	*High*	3.273[ab]	3.553	2.993	0.929[a]	1.207	0.652
			Low	0.737[b]	0.878	0.595	0.675	0.819	0.531
Proportion of time Sitting	-	209	*High*	0.024[ac]	0.084	-0.036	0.092[a]	0.153	0.031
			Low	0.275[bc]	0.357	0.193	0.096[b]	0.178	0.013
Proportion of time Standing	-	209	*High*	0.939[ac]	1.013	0.865	0.600[ad]	0.675	0.525
			Low	0.361[c]	0.458	0.263	0.408[d]	0.507	0.310
Proportion on time Lying	-	209	*High*	0.044[ab]	0.107	-0.019	0.316[ac]	0.380	0.252
			Low	0.372[b]	0.463	0.281	0.501[c]	0.593	0.408
Proportion of time Panting	-	219	*High*	0.042	0.183	0.004	0.069[a]	0.287	0.011
			Low	0.011	0.101	-0.002	0.008[a]	0.057	-0.005
Probability of Body Shake	-	231	*High*	0.776[a]	0.936	0.450	0.100[ab]	0.363	0.021
			Low	0.650	0.883	0.314	0.383[b]	0.717	0.132
Probability of Whining	-	115	*High*	-	-	-	0.189[a]	0.541	0.044
			Low	-	-	-	0.510[a]	0.816	0.541

Table S2. – Estimated means/estimated probability ($\bar{x}$) and upper/lower 95% confidence intervals (CI) for contrasts explored within models pertaining to effect of anticipated arousal and the provision of food on parameters. Matching superscript letters within a parameter indicate significant difference in means based on model contrasts (α=0.010).

				**Food Provision**					
				Food			*No Food*		
**Parameter**	**Unit**	**n**	**Arousal**	$\bar{x}$	***Upper 95% CI***	***Lower 95% CI***	$\bar{x}$	***Upper 95% CI***	***Lower 95% CI***
Cortisol	*ng/ml*	203	*High*	23.920	28.017	20.421	24.000	28.136	20.472
			Low	25.412	29.795	21.673	26.156	30.643	22.325
Heart Rate	*bpm*	188	*High*	138.548[b]	152.534	124.563	134.326[c]	148.363	120.289
			Low	125.357[ab]	139.352	111.362	108.455[ac]	122.445	94.465
Heart Rate Variability (RMSSD)	ms	191	*High*	126.039	215.680	73.652	128.756	220.790	75.084
			Low	94.003	164.271	53.791	126.194	219.850	72.434
PC1_Valence	-	234	*High*	2.420[b]	2.616	2.224	2.182[c]	2.379	1.984
			Low	1.705[ab]	2.071	1.340	0.962[ac]	1.330	0.593
PC2_Arousal	-	234	*High*	1.257[ac]	1.586	0.927	0.969[ad]	1.299	0.638
			Low	-0.556[bc]	-0.152	-0.959	-1.073[bd]	-0.667	-1.478
Serotonin	ng/mL	202	*High*	1062.701	1538.793	733.907	1024.325	1483.323	707.358
			Low	1088.325	1574.593	752.226	1036.464	1499.318	716.497
ACTH	pg/mL	201	*High*	15.293	26.496	8.825	16.373	28.376	9.445
			Low	15.125	26.420	8.657	17.809	31.090	10.200
sIgA	µg/mL	205	*High*	1190.455	1696.790	835.213	1423.689[b]	2032.020	997.475
			Low	1334.390[a]	1919.092	927.832	2175.169[ab]	3137.266	1508.115
Heart Rate Variability (SDRR)	ms	191	*High*	134.311	196.461	72.162	155.622	218.53	92.713
			Low	129.486	190.952	68.019	136.027	197.008	75.046
Left Eye Mean Temp	°C	222	*High*	35.584	36.213	34.956	35.597	36.222	34.971
			Low	35.473	36.103	34.843	35.416	36.040	34.793
Right Eye Mean Temp	°C	222	*High*	35.578	36.174	34.983	35.584	36.177	34.991
			Low	35.559	36.159	34.959	35.419	36.014	34.825
Eye Mean Temp Difference	°C	222	*High*	-0.003	0.108	-0.115	-0.010	0.100	-0.121
			Low	0.088	0.193	-0.017	0.004	0.107	-0.098
Left Ear Temp	°C	228	*High*	38.852[b]	39.110	38.595	38.812[c]	39.066	38.557
			Low	38.591[ab]	38.839	38.342	38.280[ac]	38.514	38.045
Right Ear Temp	°C	227	*High*	38.769[a]	39.032	38.507	38.766[b]	39.026	38.507
			Low	38.449[a]	38.701	38.197	38.349[b]	38.586	38.113
Ear Temp Difference	°C	226	*High*	-0.059	0.048	-0.166	-0.022	0.087	-0.130
			Low	-0.132	0.030	-0.294	0.090	0.250	-0.069
Nose Mean Temp	°C	222	*High*	26.382[a]	28.119	24.645	26.277[b]	28.003	24.551
			Low	24.323[a]	26.059	22.587	24.200[b]	25.909	22.491
Mean Whistle Activity Points	-	219	*High*	3.108[b]	3.427	2.789	3.269[c]	3.588	2.950
			Low	1.366[ab]	1.499	1.232	0.737[ac]	0.872	0.603

Proportion of Sitting	-	217	*High*	0.009	0.028	-0.011	0.018[b]	0.037	-0.001
			Low	0.044[a]	0.106	-0.017	0.266[ab]	0.329	0.203
Proportion of Standing	-	217	*High*	0.964[b]	1.024	0.904	0.938[c]	0.998	0.878
			Low	0.708[ab]	0.803	0.613	0.354[ac]	0.451	0.257
Proportion on Lying	-	217	*High*	0.028[a]	0.072	-0.016	0.044[b]	0.088	0.000
			Low	0.248[a]	0.339	0.158	0.380[b]	0.472	0.287
Proportion of Panting	-	226	*High*	0.017[a]	0.067	-0.001	0.045[a]	0.147	0.010
			Low	0.012	0.052	-0.002	0.021	0.080	0.001
Probability of Body Shake	-	234	*High*	0.318[ab]	0.682	0.092	0.798[a]	0.949	0.456
			Low	0.712[b]	0.919	0.352	0.669	0.902	0.308
